# Supplementary material for: Understanding symptom appraisal and help-seeking in people with symptoms suggestive of pancreatic cancer: a qualitative study
Source: BMJ Open. 2017 Sep 3;7(9):e015682. doi: 10.1136/bmjopen-2016-015682 (PMC5588944; doi:10.1136/bmjopen-2016-015682)
Supplement: Supplementary file 1 [file bmjopen-2016-015682supp001.pdf]

The SYMPTOM Study

Interview Topic Guide

1. Welcome and introduction

2. Confirm understanding of study, participation & recording, confidentiality issues & consent

3. Can you tell me about what first prompted you to go to your doctor?

4. Can you describe the symptom(s) a bit more?
   [Prompts: location, duration, severity, ever had this before?]

5. You mentioned (symptom(s)), were there any more symptoms that you noticed about this time?
   [Prompt: try to identify all participant-reported symptoms first; the checklist comes later]

6. What did you do to cope with/help these symptoms?
   [Prompts: How long did it take before you decided to use any treatment? How long did you try it for? Did it help at all?]

7. So, please could you help me try and record the number and chronology/timing of each symptom?
   a. *Using the Calendar landmarking tool (see below)*
   b. *By drawing a timeline of each symptom with the participant*
   c. *By using the model (attached) to identify appraisal, help-seeking, and diagnostic intervals.*

7. Now, we've talked about the symptoms that you have described: I'd also like to check whether you have had any of the following symptoms?
   [Prompt for checklist, and try to draw the timeline for each symptom: Lung symptoms: e.g. coughing up blood, dry/productive cough, chest tightness/pain, hoarse voice etc.; Colorectal & pancreas symptoms: e.g. abdominal pain, change of bowel habit, rectal bleeding, yellow jaundice etc.; General symptoms: e.g. increased fatigue, loss of appetite, weight loss etc.]

9. You went to see your doctor about your symptoms: did you have any thoughts about what might have been wrong?

10. Did you discuss your symptoms with anyone other than your Doctor?
    [Prompts: Who were they? Did they encourage/discourage you to seek medical advice?]

11. Do you know of anyone who has also had any similar symptoms or investigations to you?
    [Prompts: relationship, symptoms they had, care they received, outcome etc]

12. Can you tell me about the first appointment with your doctor for these symptoms we've been talking about?
    [Prompts: Was there a specific event that made you make the appointment? What was the trigger? Had you waited for any reason?]

13. What was discussed at that first appointment?
    [Prompts: Did you have any thoughts about what would happen when you first went to see your GP? What happened– were you examined? Did the GP arrange any tests or referrals?]

14. How did you feel about the outcome of the consultation?
    [Prompt: did you feel you were taken seriously by the GP?]

15. Did you see the GP for this symptom more than once before you were referred to the

hospital?
[Prompt: each visit may need unpacking for symptoms discussed, feelings about the consultation outcomes etc.]

16. In your opinion, do you feel there was anything that prolonged you finding out about what is causing your symptoms [*Prompts: information about the symptoms associated with serious conditions, difficulty in getting an appointment, time stresses from work/family etc., medical staff attitudes etc.]*

17. Have you experienced any symptoms or feelings or sensations that you *didn't* talk to the GP about, even if you just felt "a bit off".
[Prompt: Was there a reason why you did not mention this to your doctor?]

18. Now we've discussed your symptoms, I'd like to ask you a few questions about being referred and the test[s] you've had. Thinking back to your first consultation with your doctor, can you tell me what tests or investigations were mentioned (CXR, colonoscopy etc). When you were referred for the test can you tell me-?

A. What information the GP provided about this test
   - *[Prompts: info provided – face to face, info leaflet, website]*

B. Did you understand why you were being referred/tested and what it involved?
   - *[Prompts: knowledge of physical aspects of test, possible side effects, meaning of results - diagnosis, necessity of further tests]*

C. Do you feel that you made a conscious decision to have this test or do you feel that the decision was made for you by the GP
   - *[Prompts: feel involved in decision to be tested, conscious decision – why?]*
   - *[Prompts: accepted referral – why?; Conscious decision – why?]*
   - *[Prompts: consider not having test?]*
   - *[Prompts: discuss test with family/friends – did this influence?]*

D. How did you feel about being referred for this test
   - *[Prompts: advantages - improved prognosis, wider treatment options, reassurance; disadvantages - physical & emotional challenges, practicalities, any other aspects]*

E. Can you tell us what happened during this test
   - *[Prompts: Did you take a family member/ friend with you? what happened after, how information was communicated, how test differed from expectations]*

F. Can you tell us about the practicalities of taking this test?
   - *[Prompts: time off work – how long, manage family commitments & childcare]*

G. Do you think that all patients with symptoms like yours should have these tests?

19. We've been through a lot today but is there anything else you'd like to discuss?

20. Thanks, confirmation of consent and confidentiality.
