# Supplementary material for: Understanding symptom appraisal and help-seeking in people with symptoms suggestive of pancreatic cancer: a qualitative study
Source: BMJ Open. 2017 Sep 3;7(9):e015682. doi: 10.1136/bmjopen-2016-015682 (PMC5588944; doi:10.1136/bmjopen-2016-015682)
Supplement: Author's manuscript [file bmjopen-2016-015682.draft_revisions.pdf]

BMJ Open is committed to open peer review. As part of this commitment we make the peer review history of every article we publish publicly available.

When an article is published we post the peer reviewers' comments and the authors' responses online. We also post the versions of the paper that were used during peer review. These are the versions that the peer review comments apply to.

The versions of the paper that follow are the versions that were submitted during the peer review process. They are not the versions of record or the final published versions. They should not be cited or distributed as the published version of this manuscript.

BMJ Open is an open access journal and the full, final, typeset and author-corrected version of record of the manuscript is available on our site with no access controls, subscription charges or pay-per-view fees (http://bmjopen.bmj.com).

If you have any questions on BMJ Open's open peer review process please email editorial.bmjopen@bmj.com

# Understanding symptom appraisal and help-seeking in people with symptoms suggestive of pancreatic cancer: a qualitative study.


| | |
|---|---|
| Journal: | *BMJ Open* |
| Manuscript ID | bmjopen-2016-015682 |
| Article Type: | Research |
| Date Submitted by the Author: | 03-Jan-2017 |



| | |
|---|---|
| Complete List of Authors: | Mills, Katie; University of Cambridge, Public Health & Primary Care<br>Birt, Linda; University of East Anglia, School of Health Sciences<br>Emery, Jon; University of Melbourne, General Practice and Primary Care Academic Centre<br>Hall, Nicola; Durham University School of Medicine Pharmacy and Health<br>Banks, Jonathan; University of Bristol, Social & Community Medicine<br>Johnson, Margaret ; University of Cambridge, Patient Representative c/o Public Health & Primary Care<br>Ballard, Sue; University of Cambridge, Patient Representative c/o Public Health & Primary Care<br>Lancaster, John; University of Cambridge, Patient Representative c/o Public Health & Primary Care<br>Hamilton, Willie; University of Exeter Medical School, Primary Care Diagnostics<br>Rubin, Greg; Durham University, School of Medicine and Health<br>Walter, Fiona; University of Cambridge, Dept of Public Health and Primary Care |



| | |
|---|---|
| <b>Primary Subject Heading</b>: | Qualitative research |
| Secondary Subject Heading: | General practice / Family practice, Gastroenterology and hepatology |
| Keywords: | pancreatic cancer, diagnosis, appraisal, help-seeking, symptoms |


SCHOLARONE™ Manuscripts

# Understanding symptom appraisal and help-seeking in people with symptoms suggestive of pancreatic cancer: a qualitative study.

**Author names, affiliations, full address and postcode, contact details:**

**Katie Mills (Corresponding author**- ko298@medschl.cam.ac.uk) Tel: 01223746867
The Primary Care Unit
Department of Public Health and Primary Care
University of Cambridge
Cambridge CB1 8RN

**Linda Birt** Linda.Birt@uea.ac.uk Tel: 01603 59 3298
At the time of completing this research:
The Primary Care Unit
Department of Public Health and Primary Care
University of Cambridge
Cambridge CB1 8RN

**Jon D Emery** jon.emery@unimelb.edu.au
Department of General Practice
University of Melbourne
Australia

**Nicola Hall** n.hall@sunderland.ac.uk Tel no: 0191 515 3261
At the time of completing this research:
School of Medicine, Pharmacy & Health
Durham University
Stockton on Tees TS17 6BH

**Jonathan Banks** Jon.Banks@bristol.ac.uk
School of Social and Community Medicine
University of Bristol
Bristol BS8 2PS

**Margaret Johnson**
Patient representative c/o: ottersriverbank@natsirt.co.uk
Primary Care Unit
Department of Public Health & Primary Care
University of Cambridge
Cambridge CB1 8RN



For peer review only - http://bmjopen.bmj.com/site/about/guidelines.xhtml

**Sue Ballard** sue@ballard66.fsnet.co.uk
Patient representative c/o:
Primary Care Unit
Department of Public Health & Primary Care
University of Cambridge
Cambridge CB1 8RN

**John Lancaster** lancaster366@btinternet.com
Patient representative c/o:
Primary Care Unit
Department of Public Health & Primary Care
University of Cambridge
Cambridge CB1 8RN

**William Hamilton** W.Hamilton@exeter.ac.uk Tel no: 01392 726097
College House, St Luke's Campus
University of Exeter
Exeter EX2 4TE

**Greg P Rubin** g.p.rubin@durham.ac.uk Tel: 0191 3340031
School of Medicine, Pharmacy & Health
Durham University
Stockton on Tees TS17 6BH

**Fiona M Walter** fmw22@medschl.cam.ac.uk Tel: 01223762514
Primary Care Unit
Department of Public Health & Primary Care
University of Cambridge
Cambridge CB1 8RN

**Word count**: 4044, Tables:4

**Keywords**: pancreatic cancer, diagnosis, appraisal, help-seeking, symptoms

For peer review only

## ABSTRACT

**Objective**

Pancreatic cancer has poor survival rates, due to non-specific symptoms leading to later diagnosis. Understanding how patients interpret their symptoms could inform approaches to earlier diagnosis.

This study sought to explore symptom appraisal and help-seeking among patients referred to secondary care for symptoms suspicious of pancreatic cancer.

**Design**

Qualitative analysis of semi-structured in depth- interviews

**Participants and setting**

Patients were identified via participation in a questionnaire study after being referred to hospital with symptoms suspicious of pancreatic cancer. In-depth, semi-structured interviews were conducted soon after referral, and where possible, before diagnosis. Data were analysed iteratively and thematically, informed by the Model of Pathways to Treatment.

**Results**

26 interviews were conducted (age range 48-84 years, 14 women). Time from first symptoms to first presentation to healthcare ranged from 1 to 270 days, median 21 days. Symptom appraisal usually began with intermittent and non-specific symptoms such as tiredness or change in appetite, which developed over time, and was influenced by existing conditions, medication, family and friends. People often attributed symptoms to dietary choices or an acute illness, prompting changes in type and frequency of meals. Increasingly frequent or severe symptoms, or the development of new symptoms, usually prompted further appraisal and alternative explanations. Triggers to seek help included a change or worsening of symptoms particularly pain which was often a 'tipping point'. Failure of self-management strategies or a growing impact of symptoms on daily life also led to help-seeking. We found no differences in symptom appraisal and help seeking between people diagnosed with cancer and those with other conditions.

**Conclusions**

Greater public and healthcare professional awareness of the combinations of subtle and intermittent symptoms, and their evolving nature, is needed to prompt timelier help-seeking and investigation among people with symptoms of pancreatic cancer.




For peer review only - http://bmjopen.bmj.com/site/about/guidelines.xhtml

**STRENGTHS AND LIMITATIONS OF THIS STUDY**

- We believe this is the first study to compare the symptom appraisal and help-seeking experiences of patients referred to secondary care with symptoms suspicious of pancreatic cancer between people subsequently diagnosed with pancreatic cancer and people diagnosed with other non-cancer conditions. We were unable to identify any differences.
- The data collection and analysis of this study were guided by the model of pathways to treatment as recommended by the Aarhus Statement to improve the design and reporting of studies on early cancer diagnosis.
- Risks of recall bias and post hoc rationalisation were reduced by recruiting at the time of referral to specialist care, and interviewing patients before or close to diagnosis.
- The subtlety of initial symptoms and their evolution over time is a novel finding, and provides some understanding of the complexities faced by patients when appraising their symptoms and seeking help in a timely way.

**INTRODUCTION**

Pancreatic cancer is the ninth most common cancer in the UK, with about 8,000 cases diagnosed every year. Only 22% men and 20% women currently survive pancreatic cancer for one year or more, and it has the poorest 5-year survival rates of all cancers at less than 4% [1]. This poor prognosis is mainly due to patients being diagnosed at a stage when curative treatment is not possible. Survival could be improved if patients could be diagnosed earlier [2,3].

The key symptoms of pancreatic cancer have primarily been described from studies of patients after a diagnosis has been made. Pre-diagnosis case-control studies from the US [4] and UK [5,6] confirm that these symptoms are also relevant for the community when making healthcare management decisions. While jaundice is the only symptom which is strongly predictive of pancreatic cancer, a number of non-specific symptoms such as nausea, vomiting, abdominal pain, back pain, constipation, diarrhoea, weight loss and malaise are also weakly associated. The non-specific nature of these symptoms creates challenges for early recognition of a potentially serious condition by both patients and their GPs. In a large English database study, over 40% of patients diagnosed with pancreatic cancer had visited their GP at least three times before referral to specialist care [7].

There is limited evidence about the evolution of symptoms of pancreatic cancer over time, and how patients appraise their symptoms and decide to seek medical advice. The few studies to date have interviewed patients or their relatives many months after diagnosis [8]; such studies may be biased by *post hoc* rationalisation and recall bias. The aim of this study was therefore to explore symptom appraisal and help-seeking decisions among patients with symptoms suggestive of pancreatic cancer



For peer review only - http://bmjopen.bmj.com/site/about/guidelines.xhtml

much earlier in their diagnostic pathway. We recruited patients who were newly referred to hospital, thus providing the opportunity to investigate the complexities of patients' symptom appraisal and decision making, and explore pathways from their first symptom to first presentation in primary care, emergency presentations, and referrals to specialist services.

## METHODS

### Setting

This in-depth, face-to-face interview study was nested within the SYMPTOM Pancreas Study, a prospective cohort study investigating associations between symptoms and other factors on the total diagnostic interval and stage of diagnosis among patients with symptoms suspicious of pancreatic cancer [9]. The patients were recruited from nine hospitals in two regions of England (North East and Eastern), and similar studies were conducted for people with symptoms suggestive of lung and colorectal cancer [10-12]. Ethical approval for all the SYMPTOM studies was obtained from Cambridgeshire 3 Research Ethics Committee (10/H0303/50).

### Recruitment

Patients were recruited when referred to hospital via routine or urgent (two-week wait, '2WW') routes to a gastroenterology or hepato-pancreato-biliary (HPB) clinic or relevant ultrasound department, or admitted via A&E or other specialists. Patients were eligible if they were aged 40 years and over, and their GPs had reported symptoms suggestive of pancreatic cancer. They were initially sent an invitation letter and SYMPTOM study questionnaire by post; people who completed the questionnaire were also invited to take part in an interview. We purposively sampled patients (by region, age, gender, diagnosis) to obtain participants with a range of demographic and diagnosis characteristics.

### Data collection

Interviews were undertaken between March 2012 and March 2014 by KM, LB and NH. They took place as soon as possible after referral to hospital. All diagnoses were confirmed from review of secondary care medical records.

Before starting the interview, the researcher explained the research process and written consent was obtained. The interview was semi-structured, with the schedule developed from similar interviews undertaken with people recently diagnosed with cancer [13]. By using open-ended questions we were able to explore the patient's appraisal of their symptoms and decisions to seek help. We encouraged the interviewees to share how they made sense of their symptoms and why they chose to seek help in the context of previous experiences and existing medical conditions. To assist in the clarification of the sequences of events and key dates along the



For peer review only - http://bmjopen.bmj.com/site/about/guidelines.xhtml

pathway to diagnosis, we used a specifically designed calendar-landmarking instrument [14].

The interviews lasted between 40-70 minutes and were conducted in participants' homes. A relative, usually a spouse, was also present at several interviews at the participant's request: they assisted in recall of events and confirmed participants' accounts. Interviews continued until no new themes were identified in three consecutive interviews and data saturation was reached [15]. All interviews were audio-recorded, then professionally transcribed verbatim and anonymised via a confidential service.

**Analyses**

***Theoretical model***

Both data collection and analysis were underpinned by the theoretical approach of the Model of Pathways to Treatment [16,17] as recommended by the Aarhus Statement to improve the design and reporting of studies on early cancer diagnosis [18]. The Model considers the contributions of disease, patient and healthcare system factors to four intervals: Appraisal, Help-seeking, Diagnostic and Pre-Treatment. The first event is the detection of initial bodily changes, described as the "time point when a person becomes aware of body sensations or visual alterations, regardless of the meaning assigned to the change" [16], these then develop over time to "symptoms", defined as a bodily change perceived to be abnormal. Symptom appraisal leads to perceiving a reason to seek help, and consulting with a healthcare professional. These key events represent the "time to presentation" (TTP) period. We defined TTP as the interval between the patient-reported date of first noticing a symptom and their first consultation with a healthcare professional.

***Analytic processes***

The analytic process was iterative, starting after the first few interviews. We (KM, LB, NH) applied the Framework approach to ensure a rigorous and systematic progression through the five analytic steps: familiarisation with data, developing a thematic framework; indexing data to frame-work; mapping and questioning the data; and theoretical interpretation [19]. The Model was used at several points in the analysis process: first, to provide structure to understanding the patient pathways to diagnosis; second, to facilitate comparisons between participants diagnosed with cancer and those who presented with similar symptoms but were diagnosed with non-cancer conditions. Finally, we examined patient data by characteristics, including age, gender, co-morbidity, geographical region and diagnosis, to seek patterns and non-confirming cases. Our study patient representatives (MJ, JL) contributed to all stages of analysis, and all authors contributed to the interpretation of the data. Data management was assisted by NVivo version 9.



For peer review only - http://bmjopen.bmj.com/site/about/guidelines.xhtml

## RESULTS

### Sample

302/407 (74%) of the participants recruited into the main SYMPTOM Pancreas Study expressed an interest in an interview. 34 individuals were recruited following purposive sampling, but 8 were not interviewed as they were too unwell to participate Seven were unaware of their diagnosis at interview.

**Table 1: Participant characteristics**

| | n=26 (%) | SYMPTOM Pancreas Study n= 391 (%) |
|---|---|---|
| **English region** | | |
| Eastern | 19 (73) | 372 (95) |
| North Eastern | 7 (27) | 19 (5) |
| **Gender** | | |
| Female | 14 (54) | 196 (50) |
| Male | 12 (46) | 195 (50) |
| **Age** | | |
| Mean Years (range) | 67 (48-84) | 69 (40-93) |
| 40-59 | 7 (27) | 89 (23) |
| 60-69 | 8 (31) | 111 (28) |
| 70-79 | 6 (23) | 127 (32) |
| 80+ | 5(19) | 64 (16) |
| **Ethnicity** | | |
| White British | 26 (100) | 384 (98) |
| **Educational qualifications** | | |
| Higher Education | 4 (15) | 145 (37) |
| Further Education/Secondary school | 14 (54) | 127 (33) |
| None | 8 (31) | 119 (30) |
| **IMD quintiles** | | |
| Least deprived 1 | 6 (23) | 125 (32) |
| 2 | 8 (31) | 133 (34) |
| 3 | 5 (19) | 71 (18) |
| 4 | 5 (19) | 43 (11) |
| Most deprived 5 | 2 (8) | 16 (4) |
| Missing | - | 3 (1) |
| **Diagnoses** | | |
| **Cancer** | | |
| Pancreas | 9 | 119 |
| Duodenal | 1 | 4 |
| Gallbladder | 1 | 11 |
| Oesophageal | 1 | 12 |
| Lymphoma | 1 | 7 |
| **Other conditions** | | |
| Hiatus Hernia | 3 | 28 |
| Gallstones | 2 | 25 |
| Pancreatitis | 2 | 9 |
| Others | 6 | 75 |
| **GP appointments made** | | |
| 0* | 2 (8) | |
| 1 | 15 (58) | |
| 2 | 5 (19) | |
| 3 or more | 4 (15) | |

*emergency presentation

For peer review only



For peer review only - http://bmjopen.bmj.com/site/about/guidelines.xhtml

50% (13/26) of the interviewees were ultimately diagnosed with cancer, 9 with pancreatic cancer (3 early stage, 3 late stage, 3 unstaged), and 4 with other cancers (duodenal, gallbladder, oesophageal, lymphoma). The 13 participants with non-cancer diagnoses included hiatus hernia; gallstones and pancreatitis. All participants were interviewed soon after their referral to specialist care (range: 1 day to 18 weeks), with 6 (23%) prior to diagnosis, and a further 12 (46%) within 4 weeks of diagnosis (Table 1).

**Duration of symptoms**

Most participants were able to recall when they first noticed their symptom/s and the date they went to their GP, though for some it was difficult to recall the exact date they decided to seek help. The TTP ranged from 1-270 days, with a median of 21 days. Three individuals were frequent attenders to their GP and so we were unable to define the precise first date of presentation. Two thirds (68%) consulted their GP within 6 weeks of their trigger symptom. While more than half (58%) consulted their GPs only once before referral to secondary care, four patients (16%) saw their GP on more than three occasions.

**Qualitative themes**

We were unable to find any clear differences regarding symptom appraisal or help-seeking between the accounts of people diagnosed with cancer and those with other conditions. We therefore report the findings together. Extracts from interviews illustrate the findings: each quotation is contextualised, by the patient's gender (M/F), age (40-59, 60-69, 70-79, 80+), TTP (weeks), and diagnosis (cancer, non-cancer).

**(1) Initial appraisal of symptoms**

Participants described how they first noticed and initially interpreted their symptoms and often attributed them to a common non-serious cause or condition.

***Initial bodily changes***

People mainly described initial subtle or non-specific bodily changes such as feeling tired or 'different'. These changes were often intermittent and insidious in their development.

*"I've had this sort of tiredness and a little bit of discomfort on and off for a time"* (M, 70-79, 13-26 weeks, cancer)

*"I've felt a bit like as if I've got a nervous tummy, like it gurgles up…"* (F 50-59, regular attender to GP, non-cancer)

People also described subtle changes in appetite: *"Well I went off my food a bit I think, my daughter said I went off my food a bit"* (F, 80+yrs, 2-4 weeks, cancer)



For peer review only - http://bmjopen.bmj.com/site/about/guidelines.xhtml

***Symptom attributions (Table 2)***

Initial explanations for these bodily changes or symptoms mainly related to diet and other daily activities. Those with other conditions, particularly gastro-intestinal conditions such as gastro-oesophageal reflux disease, often appraised their new symptoms against their "usual" symptoms, and whether their treatment was still effective. This group tended to downplay their symptoms. Some people also attributed these bodily changes or symptoms to other medical conditions or side-effects of their medication. Others 'normalised' their initial symptoms to being caused by aging, common conditions such as a virus or common cold, indigestion or a urine infection.

**Table 2: Illustrative quotations relating to initial symptom attribution**

| Theme | Illustrative quotations |
|---|---|
| Diet and lifestyle | *"It comes and goes, it must be something that I eat and it just doesn't agree with me"* (F, 60-69, 13-26 weeks, non-cancer)<br><br>*"I think it's the picking up, it's the bending that does it a lot with my job as well, 'cos I lift heavy (clothing) all day, and I'm putting them over people and then bending and sitting down"* (F, 40-59, regular attender to GP, cancer) |
| Existing gastrointestinal conditions | *"I've always suffered with what's it called, you know, reflux, over the years, sometimes I could take some indigestion remedy and that Gaviscon thing and it'd calm down*" (F, 40-49, <2 weeks, non-cancer) |
| Other medical conditions, side-effects of medication | *"At first I started putting it down to the diabetic pills, I thought 'Well it's probably because I've changed those', you know, and this Pioglitazone, I kept thinking 'Well it's that',* (F 60-69, 2-4 weeks, cancer)<br><br>*"I've had this sort of tiredness and a little bit of discomfort on and off for a time, I thought it was possibly diabetes…it kept on… and we thought was perhaps as a result of some of the medication I was taking for other things."* (M, 70-79, 13-26 weeks, cancer) |
| Normalisation | *"Like most people. I think we were getting towards Christmas and I was starting to get a problem with indigestion"* (M 60-69, 5-8 weeks, non-cancer)<br><br>*"Normally it was clear, then it went, like, tea, tea-coloured, you know, right reddish brownish colour, so I says, well, there's something there…I assumed I had a urine infection"* (M, 80-89, <2 weeks, non-cancer) |

These explanations were described in the rich context of people's lives, such as being busy, caring for others, or the time of the year. When considered in these contexts, most people initially felt that their symptoms were acceptable and did not warrant seeking help:



For peer review only - http://bmjopen.bmj.com/site/about/guidelines.xhtml

*"I thought I was a bit tired-er than normal, but I mean, I was tearing around. My daughter was coming back for three months and they've got a house in the next village, I was over there doing things in the house, getting it ready."* (F, 60-69, 2-4 weeks, cancer)

***Lifestyle adaptations***

In the light of these attributions and explanations, some people began to make adaptations to their diet or daily living activities to alleviate their symptoms; in some cases, these changes were quite significant:

*"If I had smaller meals, but slightly more frequently, I could actually control keeping food, you know, keeping comfortable"* (M, 60-69, 5-8 weeks, cancer)

*"I think you learn to live with it and you just adapt to it and start to make the changes so it doesn't happen…. I think what had happened with me is I couldn't control it any longer, no matter if I stopped eating things, didn't eat out, it was happening anyway"* (F, 60-69, 26-52 weeks, non-cancer)

***Role of others in appraisal and adaptations***

Some people discussed their early symptoms with close relatives, particularly partners or children. This could prompt discussions around potential explanations and suggestions for improving symptoms, such as changes in content or timing of meals; at times it could also lead to delays in helpseeking:

*"When I mentioned to my wife the colour of my stools, which was the first symptom, she said I wasn't getting enough carbohydrates or protein or something…something wasn't right in my diet. So I changed my diet, I was eating fruit and vegetables and things like that, a lot more, and it still stayed the same."* (M, 50-59, 13-26 weeks, cancer)

**(2) Further appraisal of symptoms**

Changes in patterns of symptoms often led patients to further appraise their symptoms, and to question the reasons for their occurrence and their original explanations.

***Symptom progression (Table 3)***

Symptoms became of greater concern when they became more severe or frequent, or when further subtle symptoms developed. In some cases, it appeared that there was progression from subtle and intermittent symptoms of a manageable and tolerable nature, to symptoms which were more alarming such as pain. Across the whole sample, people described pain in a number of different parts of the body including their shoulder, chest, upper abdomen and back.

Another man described the chronology of his symptoms; having started with mild indigestion symptoms, additional, more alarming, symptoms developed: *"gradually… the stools over a period of time, but the urine it seemed to happen more or less in a week, from normal colour to really deep colour"* (M, 50-59, 13-26 weeks, cancer)

Many participants reported subtle yet progressive changes to their appetite and enjoyment of food. Weight loss as a result of changes in appetite was also reported by a few participants.



For peer review only - http://bmjopen.bmj.com/site/about/guidelines.xhtml

**Table 3: Illustrative quotations relating to further appraisal of symptom progression**

| Theme | Illustrative quotations |
|---|---|
| Pain | *"Well, it varied, you know, sometimes I'd have the pain all day and that's very wearing and painful…It's just under the rib cage and it's slightly to the right hand side, just slightly there, always in the same place."* (F, 70-79, <2 weeks, non-cancer)<br><br>*"…I just had a nagging, more like a dull ache, underneath my bust and I thought it was indigestion and it didn't seem to go away, it didn't make any difference whether I ate or not, it was there quite a lot of the time"* (F, 70-79, 2-4 weeks, cancer)<br><br>*"After Christmas that then became all day, every day. I was going to bed with a stomach ache and I was waking up with a stomach ache…It was there all the while and you know, it didn't make sense that I'd have a problem all the while so I thought I was imagining it"* (M, 60-69, 2-4 weeks, cancer) |
| Progressive changes in appetite and enjoyment of food | *"I was actually going off the actual taste of food, I wasn't enjoying food anymore, things that I would normally eat I wasn't enjoying"* (M, 60-69, 5-8 weeks, cancer)<br><br>*"Well it became quite traumatic at one point, when I just [did] not want an evening meal at all. I always eat a reasonable breakfast, but for the evening meal I had no appetite"* (M, 80+, <2 weeks, cancer)<br><br>*… and I was eating less and smaller meals and being really careful what I ate, and I thought "it's good for you but you shouldn't have to be that precise when you eat"* (F, 60-69, 26-52 weeks, non-cancer) |
| Weight loss | *"I was also suffering uncomfortable, you know, let's say stomach irritation, indigestion, wasn't happy eating, so I literally went along to say 'look, this isn't right, I don't mind losing weight but not like this"* (M 60-69, 5-8 weeks, cancer)<br><br>*"I'd probably lost about four pounds which was concerning me but then I thought, you've reduced the amount that you're eating…"* (F 50-59, 2-4 weeks, non-cancer) |

For peer review only

***Symptom monitoring and self-management***

The subtlety and slow progression of symptoms prompted some participants to monitor their symptoms, and some to consider alternative self-management strategies. Many used over-the-counter remedies to attempt to alleviate their symptoms of indigestion or abdominal pain:

*"It was only when I started feeling a bit of strangeness that I decided to try and keep a pattern of them"* (F, 60-69, 2-4 weeks, cancer)



For peer review only - http://bmjopen.bmj.com/site/about/guidelines.xhtml

***Impact on life***

Most people described how their symptoms gradually or rapidly impacted on their quality of life and daily routine, including their social activities and hobbies, as well as their ability to sleep. Some felt that they were less able to complete tasks at home; for others, work became more challenging:

*"I decided I'd got this burning pain and I noticed that I was beginning to get a little bit short of breath, and also I couldn't do the same things I used to, like I found digging was becoming impossible"* (M, 70-79, 13-26 weeks, non-cancer)

***Previous family and personal history of cancer***

A few people expressed a concern that they might develop cancer because they had lost a close relative to the disease, or suffered from cancer themselves. These people were concerned about their personal risk of cancer and reported that they often considered whether their symptoms matched those which they expected from having cancer. Conversely one woman, subsequently diagnosed with a benign condition, had not considered cancer as a possibility because her symptoms did not match her expectations of cancer:

*"I knew I'm generally healthy, I mean I've been going swimming and to the gym…it wasn't seriously debilitating…I expect when you have cancer that you have more symptoms"* (F, 60-69, 26-52 weeks, non-cancer)

**(3) Deciding to seek help (Table 4)**

The further appraisal of symptoms eventually led most participants to decide to seek help from primary care, although several had emergency visits to A&E. Triggers to seek help usually involved an increased frequency or intensity of symptoms, although sometimes it was precipitated by an alarming event, usually pain. Pain was often the "tipping point" to seek help against a background of other more subtle symptoms. For those without pain, triggers were often complex, and could involve interpreting new or changing symptoms alongside symptoms from other existing conditions and, in some instances, symptoms from other acute illnesses. Healthcare factors, such as availability of a GP appointment, were seldom mentioned.

People were often encouraged to seek help by their spouse, family members and friends; they were particularly influenced by comments about changes in their general wellbeing, or recall of specific symptom episodes. A few participants reported considering whether their symptoms had become a 'legitimate' reason to seek healthcare when self-management strategies became ineffective; they tended to refer to the length of time symptoms had been experienced, and their reduced ability to cope.



For peer review only - http://bmjopen.bmj.com/site/about/guidelines.xhtml

**Table 4: Illustrative quotations relating to deciding to seek help**

| Themes | Illustrative quotations |
|---|---|
| Pain on a background of more subtle symptoms | *"Sometimes I could take some indigestion remedy and.. it'd calm down, but this were a different kind of pain, this were, oh it's excruciating pain…but that were the first time that… I thought there's something not right here and I need to get some help."* (F, 40-49, <2 weeks, non-cancer) |
| Help-seeking encouraged by others | *"The kids had been saying "mum you're always ill" and I thought "oh yeah, I am". Because you, I think you sort of drift, you get used to things and you don't see any problem with it, and when other people start to say, you know "hang on" and then I thought "oh yeah"*(F, 60-69, 26-52 weeks, non-cancer)<br><br>*"I'd fall asleep on my desk, you know, this isn't me, and other people said "oh you're losing weight"… people actually were saying "you don't look well". It's another lever to go and see, to get it sorted"* (M, 60-69, 5-8 weeks, cancer) |
| Legitimate reason | *"I think my basic problem was pain… from arthritis that I'd had for some time, but it was proving impossible to control it by the methods I was using"* (M 80-89, 2-4 weeks, cancer) |

For peer review only

**DISCUSSION**

This is the first study to investigate symptom appraisal and help-seeking in patients newly referred to hospital with symptoms suspicious of pancreatic cancer. Due to their subtle and intermittent pattern, many people initially just monitored their symptoms. Symptoms were interpreted based on previous experiences with common conditions such as gastroenteritis, or impact of dietary change or existing gastro-intestinal conditions. Many made changes to their eating patterns including type of food, and frequency and quantity of meals, or managed their symptoms with over-the-counter medication. Consequently, they tended not to go to their GP at that point. Changes to symptom frequency, duration or severity, their impact on daily life, or the appearance of additional symptoms, particularly pain, led many people to further appraise their symptoms and visit their GP. Family and friends often made important contributions to encouraging help-seeking by reflecting on changes in symptoms, although at times social contacts could re-inforce self-management strategies. These patterns of symptom appraisal and help-seeking were seen regardless of final diagnosis.

**Strengths and limitations**

The major strength of this study is that we prospectively recruited patients with symptoms suggestive of pancreatic cancer, and interviewed them during their pathway to diagnosis and treatment. This allowed detailed discussions about factors affecting symptom appraisal and help-seeking while they were still fresh events, and greatly reduced the possibility of *post-hoc* rationalisation and recall bias. We have



For peer review only - http://bmjopen.bmj.com/site/about/guidelines.xhtml

used similar approaches in interview studies with people recently diagnosed with melanoma [20], and people with symptoms suggestive of lung [10] and colorectal [12] cancers. Recruiting patients during their diagnostic pathway enabled comparisons about symptom appraisal and help-seeking between people subsequently diagnosed with cancer and people with non-cancer conditions; importantly, we were not able to find differences between them. Our recruitment process meant that some patients were subsequently diagnosed with a cancer other than pancreatic cancer. We do not consider this a weakness of the study; rather, it serves to highlight the complexity and challenges of diagnosing pancreatic cancer, and how similarly a range of upper gastro-intestinal cancers can present. While we aimed to recruit equal numbers of patients with pancreatic cancer and benign conditions, several patients with cancer withdrew before interview as they were too unwell.

Our approach to data collection and analysis was strengthened by the use of a strong theoretical framework: underpinned by recommendations from the Aarhus statement on improving design and reporting studies on early cancer diagnosis [18], we applied the framework of the widely used Model of Pathways to Treatment [16,17], and a specifically-developed calendar-landmarking instrument which aided some participants' recall of dates of their symptoms and help-seeking [14]. Other strengths include recruitment from two English regions and a number of hospitals to ensure patients from a wide range of socio-economic backgrounds, and who had entered hospital care via a variety of routes, enabling us to gather a broad range of experiences. Finally, the research team had a broad range of scientific and clinical expertise, and we sought input from our lay members at all stages of the research process, including analysis and interpretation of the data.

While we acknowledge that these experiences may not be representative of all people with symptoms suggestive of pancreatic cancer, the demographics of this cohort are similar to that of the main SYMPTOM-Pancreas study. Although a major finding was that there were similar patterns in symptom appraisal and help-seeking between those diagnosed with cancer and those with other diagnoses, this may have been partly due to recruiting patients relatively late in their diagnostic pathway, when GPs had referred with a suspicion of pancreatic cancer. Ideally we would have recruited people with similar symptoms from earlier in their diagnostic pathway and from primary care, but there are significant challenges with the feasibility and costs of recruiting such a cohort containing sufficient cases of pancreatic cancer. Finally, we can only report the experiences shared with the researchers at the time of the interview: there may have been events which were not discussed if they were felt to be too emotional or private.



For peer review only - http://bmjopen.bmj.com/site/about/guidelines.xhtml

**Comparison with existing literature**

Very little has been published on symptom appraisal and help-seeking among patients diagnosed with pancreatic cancer. Evans *et al.* recently reported the only other similar study, also set in England, and highlighted the intermittent and subtle nature of symptoms with their title: '"It can't be very important because it comes and goes"- patients' accounts of intermittent symptoms preceding a pancreatic cancer diagnosis' [8]. However, their interviews were conducted with people up to several years after a diagnosis of pancreatic cancer, and included data from interviews with relatives of people who had died. Our findings are strengthened by interviewing patients earlier in their illness pathway, thereby capturing subtle assessments of initial bodily changes and explanations, and minimising post-hoc rationalisation and recall bias. We could not identify any subtle differences in the nature of symptoms or help-seeking between those who eventually were found to have pancreatic cancer, and those who were not. This is consistent with the quantitative findings of the SYMPTOM-pancreas study [9], where the evolution of additional symptoms such as fatigue, change in bowel habit, weight loss, decreased appetite and jaundice were associated with pancreatic cancer.

Other studies of pancreatic cancer symptoms have mainly used quantitative retrospective designs. For example, Stapley *et al*. undertook a case-control study using routinely recorded UK primary care data which demonstrated how poorly predictive most symptoms are of pancreatic cancer in primary care populations. While the positive predictive value of jaundice was relatively high (21%), it was much lower for other symptoms including abdominal and back pain, nausea, vomiting, change in bowel habit, weight loss and malaise (<1%) [6]. Keane *et al*. more recently used another large UK primary care database to investigate the early symptom profiles of patients diagnosed with pancreatic ductal or biliary adenocarcinoma and found a similar wide range of early symptoms [21]. They also found that, in the year prior to diagnosis, these patients visited their GP on a median of 18 (IQR 11–27) occasions, which resonated with findings from another large English database study showing that 41% of patients diagnosed with pancreatic cancer had visited their GP at least three times before referral to specialist care [7]. In contrast, more than half our sample reported only visiting their GP once before referral, suggesting either that our sample were recruited early in their symptom pathway, or perhaps that they had symptoms such as jaundice which are more likely to prompt early referral. Other quantitative studies set among patients newly diagnosed with pancreatic cancer patients have reported fatigue, dyspepsia and subtle changes in appetite such as early satiety in the preceding six months, confirming our finding that there may be a window of opportunity for timely investigations when patients report combinations of non-specific or subtle symptoms [4,22,23].



For peer review only - http://bmjopen.bmj.com/site/about/guidelines.xhtml

**Implications for research and practice**

Our findings add to current knowledge about the subtle and non-specific nature of early symptoms of pancreatic cancer, and enhance our understanding of their often intermittent and insidious nature. They highlight the importance of on-going, and often iterative, appraisal of symptoms as described in the Model of Pathways to Treatment [16,17]. The findings have characterised pain as a 'tipping point' for presentation to healthcare, and underpinned the importance of family and friends in symptom monitoring, recognising evolving and new symptoms and endorsement of help-seeking [24]. The challenge for symptom awareness campaigns is to promote earlier presentation with these more subtle and intermittent symptoms which lead to changes in eating patterns including type of food, and frequency and quantity of meals, and weight loss. The 'Know 4 Sure' campaign was one of the first to raise awareness of non-site-specific symptoms including weight loss, pain, lump and unexplained bleeding. It was associated with increase in referrals for a range of cancers including upper GI cancer [25]. Whether public health campaigns can effectively promote messages about combinations of non-specific symptoms is unknown, but may be important in pancreatic cancer.

The newly revised NICE guidance on Suspected Cancer: Recognition and Referral [26] supports a lower threshold for investigating and referring people with single and multiple symptoms suggestive of pancreatic cancer, particularly those in higher risk groups such as newly diagnosed diabetics. The findings from this study could inform GP education approaches to promote timely investigation and referral for people with these non-specific and intermittent symptoms. These numerous subtle and non-specific symptoms, when combined with abnormal blood tests and the use of clinical decision support tools, might alert GPs to a possible pancreatic cancer diagnosis earlier. Appropriate safety-netting is also important due to the evolving nature of symptoms, including symptom monitoring and early review.

In conclusion, this study has described and contextualised the appraisal and help-seeking behaviours of patients with symptoms that could represent pancreatic cancer. Greater awareness of combinations of the subtle and intermittent symptoms, and their evolving nature, is needed to prompt timelier help-seeking and investigation among people with symptoms of pancreatic cancer.



For peer review only - http://bmjopen.bmj.com/site/about/guidelines.xhtml

**Acknowledgments**

We thank the patients who so kindly and freely gave their time and experiences to this study. We thank Helen Morris the SYMPTOM study manager, our other study patient and public representative Victor Boulter, and the staff at the participating hospitals (Bedford Hospital NHS Trust, Cambridge University Hospitals NHS Foundation Trust; Hinchingbooke Healthcare NHS Trust, The Queen Elizabeth Hospital Kings Lynn NHS Trust, Peterborough and Stamford NHS Foundation Trust, University Hospital of North Durham; Darlington Memorial Hospital: University Hospital of North Tees, West Suffolk NHS Foundation Trust) who facilitated participant recruitment and supported the study. The authors would also like to acknowledge the contribution made by the NIHR funded Discovery Programme Steering Committee comprising: Roger Jones (chair); Alison Clutterbuck; Ardiana Gjini; Joanne Hartland; Maire Justice; Jenny Knowles; Richard Neal; Peter Rose.

**Contributors**

FMW, JE, GR and WH were responsible for study design, study conduct and management. KM, LB and NH collected the data, and with the support of JB, JE, JL, MJ, SB and FMW, KM analysed and interpreted the data. KM led on the manuscript preparation. All authors contributed to the writing and completion of the paper and have approved the final the manuscript.

**Funding**

This paper presents independent research funded by the National Institute for Health Research Programme Grants for Applied Research programme (RP-PG-0608-10045) and Pancreatic Cancer Action (registered charity 1137689). The views expressed are those of the authors and not necessarily those of the NHS, the NIHR or the Department of Health. The funders of this study had no role in study design, data collection, analysis, and interpretation, or the writing of this research paper. FMW is supported by an NIHR Clinician Scientist award.

**Competing interests**

The authors have no conflicts of interest to declare.

**Data sharing statement**

No additional data are available.

**Ethics approval**

Ethical approval was granted by the Cambridgeshire 3 Research Ethics Committee (10/H0306/50).



For peer review only - http://bmjopen.bmj.com/site/about/guidelines.xhtml

**REFERENCES**


1. Cancer Research UK, *Incidence Statistics* 2011. Retrieved from http://www.cancerresearchuk.org/health-professional/cancer-statistics/incidence/common-cancers-compared#heading-Zero (Last accessed 31.05.16).

2. Hiom S. Diagnosing cancer earlier: reviewing the evidence for improving cancer survival. *Br J Cancer* 2015; 112 (supp1): S1-S5.

3. Neal RD, Tharmanathan P, France B et al. Is increased time to diagnosis and treatment in symptomatic cancer associated with poorer outcomes? Systematic review. Br J Cancer 2015; 112:S92-S107.

4. Holly EA, Chaliha I, Bracci PM, et al. Signs and symptoms of pancreatic cancer: a population-based case-control study in the San Francisco Bay area. *Clin Gastroenterol Hepatol* 2004; 2:510-7.

5. Hippisley-Cox J, Coupland C. Identifying patients with suspected pancreatic cancer in primary care: derivation and validation of an algorithm. *Br J Gen Pract* 2012; 62: e38-45.

6. Stapley S, Peters TJ, Neal RD, et al. The risk of pancreatic cancer in symptomatic patients in primary care: a large case-control study using electronic records. *Br J Cancer* 2012; 106: 1940-4.

7. Lyratzopoulos G, Neal RD, Barbiere JM, et al. Variation in number of general practitioner consultations before hospital referral for cancer: findings from the 2010 National Cancer Patient Experience Survey in England, *The Lancet Oncology* 2012; 13: 353–365.

8. Evans J, Chapple A, Salisbury H, et al. "It can't be very important because it comes and goes"--patients' accounts of intermittent symptoms preceding a pancreatic cancer diagnosis: a qualitative study. *BMJ Open* 2014; 4: e004215.

9. Walter FM, Mills K, Mendonca SC, et al. Symptoms and patient factors associated with diagnostic intervals for pancreatic cancer (SYMPTOM pancreatic study): a prospective cohort study. Lancet Gastroenterol Hepatol 2016; 1:298-306.

10. Birt L, Hall N, Emery J, et al. Responding to symptoms suggestive of lung cancer: a qualitative interview study. *BMJ Open Respir Res* 2014; 1(1): e000067.

11. Walter FM, Rubin G, Bankhead C, et al. Symptoms and other factors associated with time to diagnosis and stage of lung cancer: a prospective cohort study. *Br J Cancer* 2015; 112 (supp1): S6-S13.




For peer review only - http://bmjopen.bmj.com/site/about/guidelines.xhtml

12. Hall N, Birt L, Banks J et al. Symptom appraisal and healthcare-seeking for symptoms suggestive of colorectal cancer: a qualitative study. *BMJ Open* 2015;5(10):e008448.

13. Emery JD, Walter FM, Gray V, et al. Diagnosing cancer in the bush: a mixed methods study of GP and specialist diagnostic intervals in rural Western Australia. *Fam Pract* 2013; 30(5):541-50.

14. Mills K, Emery J, Cheung C, et al. A qualitative exploration of the use of calendar landmarking instruments in cancer symptom research. BMC *Fam Pract* 2014; 15:167.

15. Guest G, Bunce A, Johnson L. How many interviews are enough? An experiment in data saturation and variability. *Field Methods* 2006; 18:59-82.

16. Walter FM, Scott SE, Webster A, et al. The Andersen Model of Total Patient Delay: a systematic review of its application in cancer diagnosis. *J Health Services Research & Policy* 2012; 17(2):110-8.

17. Scott SE, Walter FM, Webster A et al. The model of pathways to treatment: conceptualization and integration with existing theory. *Br J Health Psychol* 2013; 18(1): 45-65.

18. Weller D, Vedsted P, Rubin G, et al. The Aarhus statement: improving design and reporting of studies on early cancer diagnosis. *Br J Cancer* 2012; 106(7):1262-1267.

19. Ritchie J, Spencer L. Qualitative data analysis for applied policy research. In: Bryman A, Burgess R, eds. *Analysing qualitative data*. London: Routledge, 1993:173-94.

20. Walter FM, Birt L, Cavers D, et al. This isn't what mine looked like: a qualitative study of symptom appraisal and help-seeking in people recently diagnosed with melanoma. *BMJ Open* 2014; 21: 4(7) e005566.

21. Keane MG, Horsfall L, Rait G, Pereira SP. A case-control study comparing the incidence of early symptoms in pancreatic and biliary tract cancer. *BMJ Open* 4:e005720.

22. Gullo L, Tomassetti P, Migliori M, et al. Do early symptoms of pancreatic cancer exist that can allow an earlier diagnosis? *Pancreas* 2001; 22:210-3.

23. Ingeman ML, Christensen MB, Bro F. The Danish cancer pathway for patients with serious non-specific symptoms and signs of cancer–a cross-sectional study of patient characteristics and cancer probability, *BMC Cancer* 2015; 15:421.

24. Leydon GM, Bynoe-Sutherland J, Coleman MP. The journey towards a cancer diagnosis: the experiences of people with cancer, their family and carers. *Eur J Cancer Care* 2003; 12:317-326.

For peer review only



For peer review only - http://bmjopen.bmj.com/site/about/guidelines.xhtml

25. Cancer Research UK, "Know 4 sure" campaign 2014. Retrieved from http://www.cancerresearchuk.org/health-professional/early-diagnosis-activities/be-clear-on-cancer/know-4-sure-campaign (Last accessed 31.05.2016).

26. National Institute for Health and Care Excellence, Suspected Cancer: Recognition and Referral 2015. Retrieved from http://www.nice.org.uk/guidance/ng12 (Last accessed 31.05.16).

For peer review only



For peer review only - http://bmjopen.bmj.com/site/about/guidelines.xhtml

BMJ Open

# Understanding symptom appraisal and help-seeking in people with symptoms suggestive of pancreatic cancer: a qualitative study.

| | |
|---|---|
| Journal: | *BMJ Open* |
| Manuscript ID | bmjopen-2016-015682.R1 |
| Article Type: | Research |
| Date Submitted by the Author: | 02-May-2017 |
| Complete List of Authors: | Mills, Katie; University of Cambridge, Public Health & Primary Care<br>Birt, Linda; University of East Anglia, School of Health Sciences<br>Emery, Jon; University of Melbourne, General Practice and Primary Care Academic Centre<br>Hall, Nicola; Durham University School of Medicine Pharmacy and Health<br>Banks, Jonathan; University of Bristol, Social & Community Medicine<br>Johnson, Margaret ; University of Cambridge, Patient Representative c/o Public Health & Primary Care<br>Lancaster, John; University of Cambridge, Patient Representative c/o Public Health & Primary Care<br>Hamilton, Willie; University of Exeter Medical School, Primary Care Diagnostics<br>Rubin, Greg; Durham University, School of Medicine and Health<br>Walter, Fiona; University of Cambridge, Dept of Public Health and Primary Care |
| <b>Primary Subject Heading</b>: | Qualitative research |
| Secondary Subject Heading: | General practice / Family practice, Gastroenterology and hepatology |
| Keywords: | pancreatic cancer, diagnosis, appraisal, help-seeking, symptoms |

For peer review only

SCHOLARONE™
Manuscripts

# Understanding symptom appraisal and help-seeking in people with symptoms suggestive of pancreatic cancer: a qualitative study.

**Author names, affiliations, full address and postcode, contact details:**

**Katie Mills (Corresponding author**- ko298@medschl.cam.ac.uk)
The Primary Care Unit
Department of Public Health and Primary Care
University of Cambridge
Cambridge CB1 8RN

**Linda Birt** Linda.Birt@uea.ac.uk
At the time of completing this research:
The Primary Care Unit
Department of Public Health and Primary Care
University of Cambridge
Cambridge CB1 8RN

**Jon D Emery** jon.emery@unimelb.edu.au
Department of General Practice
University of Melbourne
Australia

**Nicola Hall** n.hall@sunderland.ac.uk
At the time of completing this research:
School of Medicine, Pharmacy & Health
Durham University
Stockton on Tees TS17 6BH

**Jonathan Banks** Jon.Banks@bristol.ac.uk
School of Social and Community Medicine
University of Bristol
Bristol BS8 2PS

**Margaret Johnson**
Patient representative c/o: ottersriverbank@natsirt.co.uk
Primary Care Unit
Department of Public Health & Primary Care
University of Cambridge
Cambridge CB1 8RN



For peer review only - http://bmjopen.bmj.com/site/about/guidelines.xhtml

**John Lancaster** lancaster366@btinternet.com
Patient representative c/o:
Primary Care Unit
Department of Public Health & Primary Care
University of Cambridge
Cambridge CB1 8RN

**William Hamilton** W.Hamilton@exeter.ac.uk
College House, St Luke's Campus
University of Exeter
Exeter EX2 4TE

**Greg P Rubin** g.p.rubin@durham.ac.uk
School of Medicine, Pharmacy & Health
Durham University
Stockton on Tees TS17 6BH

**Fiona M Walter** fmw22@medschl.cam.ac.uk
Primary Care Unit
Department of Public Health & Primary Care
University of Cambridge
Cambridge CB1 8RN

**Keywords**: pancreatic cancer, diagnosis, appraisal, help-seeking, symptoms



For peer review only - http://bmjopen.bmj.com/site/about/guidelines.xhtml

## ABSTRACT

### Objective

Pancreatic cancer has poor survival rates, due to non-specific symptoms leading to later diagnosis. Understanding how patients interpret their symptoms could inform approaches to earlier diagnosis. This study sought to explore symptom appraisal and help-seeking among patients referred to secondary care for symptoms suggestive of pancreatic cancer.

### Design

Qualitative analysis of semi-structured in-depth interviews. Data were analysed iteratively and thematically, informed by the Model of Pathways to Treatment.

### Participants and setting

Patients aged ≥ 40 years were recruited from nine hospitals after being referred to hospital with symptoms suggestive of pancreatic cancer; all were participants in a cohort study. Interviews were conducted soon after referral, and where possible, before diagnosis.

### Results

Twenty-six interviews were conducted (age range 48-84 years, 14 women). Time from first symptoms to first presentation to healthcare ranged from 1 to 270 days, median 21 days. Symptom appraisal usually began with intermittent and non-specific symptoms such as tiredness or change in appetite, which developed over time, and was influenced by existing conditions, medication, family and friends. People often attributed symptoms to dietary choices or an acute illness, prompting changes in type and frequency of meals. Increasingly frequent or severe symptoms, or the development of new symptoms, usually prompted further appraisal and alternative explanations. Triggers to seek help included a change or worsening of symptoms particularly pain which was often a 'tipping point'. Failure of self-management strategies or a growing impact of symptoms on daily life also led to help-seeking. We found no differences in symptom appraisal and help seeking between people diagnosed with cancer and those with other conditions.

### Conclusions

Greater public and healthcare professional awareness of the combinations of subtle and intermittent symptoms, and their evolving nature, is needed to prompt timelier help-seeking and investigation among people with symptoms of pancreatic cancer.


For peer review only



For peer review only - http://bmjopen.bmj.com/site/about/guidelines.xhtml

**STRENGTHS AND LIMITATIONS OF THIS STUDY**

- We believe this is the first study to compare the symptom appraisal and help-seeking experiences of patients referred to secondary care with symptoms suggestive of pancreatic cancer between people subsequently diagnosed with pancreatic cancer and people diagnosed with other non-cancer conditions. We were unable to identify any differences.

- The data collection and analysis of this study were guided by the model of pathways to treatment as recommended by the Aarhus Statement to improve the design and reporting of studies on early cancer diagnosis.

- Risks of recall bias and post hoc rationalisation were reduced by recruiting at the time of referral to specialist care, and interviewing patients before or close to diagnosis.

- The subtlety of initial symptoms and their evolution over time is a novel finding, and provides some understanding of the complexities faced by patients when appraising their symptoms and seeking help in a timely way.

- Some of the most seriously ill patients were unable to be interviewed and their experiences may have differed from those in our sample.

For peer review only



For peer review only - http://bmjopen.bmj.com/site/about/guidelines.xhtml

## INTRODUCTION

Pancreatic cancer is the ninth most common cancer in the UK, with about 8,000 cases diagnosed every year. Only 22% men and 20% women currently survive pancreatic cancer for one year or more, and it has the poorest 5-year survival rates of all cancers at less than 4% [1]. This poor prognosis is mainly due to patients being diagnosed at a stage when curative treatment is not possible. Survival could be improved if patients could be diagnosed earlier [2,3].

The key symptoms of pancreatic cancer have primarily been described from studies of patients after a diagnosis has been made. Pre-diagnosis case-control studies from the US [4] and UK [5,6] confirm that these symptoms are also relevant for the community when making healthcare management decisions. While jaundice is the only symptom which is strongly predictive of pancreatic cancer, a number of non-specific symptoms such as nausea, vomiting, abdominal pain, back pain, constipation, diarrhoea, weight loss and malaise are also weakly associated [6]. The non-specific nature of these symptoms creates challenges for early recognition of a potentially serious condition by both patients and their GPs. In a large English database study, over 40% of patients diagnosed with pancreatic cancer had visited their GP at least three times before referral to specialist care [7].

There is limited evidence about the evolution of symptoms of pancreatic cancer over time, and how patients appraise their symptoms and decide to seek medical advice. One of the few studies to date interviewed patients or their relatives many months after diagnosis [8]; such studies may be biased by *post hoc* rationalisation and recall bias. Furthermore, many symptoms of pancreatic cancer are also symptoms of other gastrointestinal or intra-abdominal cancers such as gallbladder, colon and ovarian cancer, which may be equally difficult to diagnose early [7].

The aim of this study was therefore to gain understanding of barriers and facilitators to symptom appraisal and help-seeking decisions among patients with symptoms suggestive of pancreatic cancer much earlier in their diagnostic pathway, to contribute to the development of interventions to promote timely diagnosis. We recruited patients who were newly referred to hospital, thus providing the opportunity to investigate the complexities of patients' symptom appraisal and decision making, and explore pathways from their first symptom to first presentation in primary care, emergency presentations, and referrals to specialist services.



For peer review only - http://bmjopen.bmj.com/site/about/guidelines.xhtml

## METHODS

### Setting

This in-depth, face-to-face interview study was nested within the SYMPTOM Pancreas Study, a prospective cohort study investigating associations between symptoms and other factors on the total diagnostic interval and stage of diagnosis among patients with symptoms suggestive of pancreatic cancer [9]. The patients were recruited from nine hospitals in two regions of England (North East and Eastern), and similar studies were conducted for people with symptoms suggestive of lung and colorectal cancer [10-12]. Ethical approval for all the SYMPTOM studies was obtained from Cambridgeshire 3 Research Ethics Committee (10/H0303/50).

### Recruitment

Patients were recruited when referred to hospital via routine or urgent (two-week wait, '2WW') routes to a gastroenterology or hepato-pancreato-biliary (HPB) clinic or relevant ultrasound department, or admitted via A&E or other specialists. Patients were eligible if they were aged 40 years and over, and their GPs had reported symptoms suggestive of pancreatic cancer. They were initially sent an invitation letter and SYMPTOM study questionnaire by post; people who completed the questionnaire were able to indicate whether they would be willing to take part in an interview. We purposively sampled patients (by region, age, gender, diagnosis) to obtain participants with a range of demographic and diagnosis characteristics.

### Data collection

Interviews were undertaken between March 2012 and March 2014 by KM, LB and NH. They took place as soon as possible after referral to hospital. All diagnoses were confirmed from review of secondary care medical records.

Before starting the interview, the researcher explained the research process and written consent was obtained. The interview was semi-structured, with the schedule developed from similar interviews undertaken with people recently diagnosed with cancer [13]. By using open-ended questions we were able to explore the patient's appraisal of their symptoms and decisions to seek help. We encouraged the interviewees to share how they made sense of their symptoms and why they chose to seek help in the context of previous experiences and existing medical conditions. To assist in the clarification of the sequences of events and key dates along the pathway to diagnosis, we used a specifically designed calendar-landmarking instrument [14].

The interviews lasted between 40-70 minutes and were conducted in participants' homes. A relative, usually a spouse, was also present at several interviews at the participant's request: they assisted in recall of events and confirmed participants'



For peer review only - http://bmjopen.bmj.com/site/about/guidelines.xhtml

accounts, but only the participant's words were included as data in analyses. Interviews continued until no new themes were identified in three consecutive interviews and data saturation was reached [15]. All interviews were audio-recorded, then professionally transcribed verbatim and anonymised via a confidential service.

**Analyses**

***Theoretical model***

Both data collection and analysis were underpinned by the theoretical approach of the Model of Pathways to Treatment [16,17] as recommended by the Aarhus Statement to improve the design and reporting of studies on early cancer diagnosis [18]. The Model considers the contributions of disease, patient and healthcare system factors to four intervals: Appraisal, Help-seeking, Diagnostic and Pre-Treatment. The first event is the detection of initial bodily changes, described as the "time point when a person becomes aware of body sensations or visual alterations, regardless of the meaning assigned to the change" [16], these then develop over time to "symptoms", defined as a bodily change perceived to be abnormal. Symptom appraisal leads to perceiving a reason to seek help, and consulting with a healthcare professional. These key events represent the "time to presentation" (TTP) period. We defined TTP as the interval between the patient-reported date of first noticing a symptom and their first consultation with a healthcare professional.

***Analytic processes***

The analytic process was iterative, starting after the first few interviews. Three researchers with social sciences expertise (KM, LB, NH) worked together, initially using thematic analysis techniques [19] to look for unexpected inductive codes and categories, and then adopting a framework approach [20] guided by the stages of the Model of Pathways to Treatment [16.17]. These approaches were combined to ensure a rigorous and systematic progression through the five analytic steps: familiarisation with data, developing a thematic framework; indexing data to frame-work; mapping and questioning the data; and theoretical interpretation [21]. The Model was used at several points in the analysis process: first, to provide structure to understanding the patient pathways to diagnosis; second, to facilitate comparisons between participants diagnosed with cancer and those who presented with similar symptoms but were diagnosed with non-cancer conditions. Finally, the researchers examined patient data by characteristics, including age, gender, co-morbidity, geographical region and diagnosis, to seek patterns and non-confirming cases. The study patient representatives (MJ, JL) contributed to all stages of analysis by reading transcripts and regular meetings with the research team, and the other authors (all with clinical or social science expertise) contributed to the interpretation of the data. Data management was assisted by NVivo version 9.



For peer review only - http://bmjopen.bmj.com/site/about/guidelines.xhtml

## RESULTS

### Sample (Table 1)

302/407 (74%) of the participants recruited into the main SYMPTOM Pancreas Study expressed an interest in an interview. Thirty-four individuals were recruited following purposive sampling, but 8 were not interviewed as they were too unwell to participate. Six were unaware of their diagnosis at interview.

Half (13/26) of the interviewees were ultimately diagnosed with cancer, 9 with pancreatic cancer (3 early stage, 3 late stage, 3 unstaged), and 4 with other cancers (duodenal, gallbladder, oesophageal, lymphoma). The 13 participants with non-cancer diagnoses included hiatus hernia, gallstones and pancreatitis. All participants were interviewed soon after their referral to specialist care (range: 1 day to 18 weeks), with a quarter (6, 23%) prior to diagnosis, half (12, 46%) within 4 weeks of diagnosis, and the remainder within 18 weeks (8, 31%).

### Duration of symptoms

Most participants were able to recall when they first noticed their symptom/s and the date they went to their GP, though for some it was difficult to recall the exact date they decided to seek help. The TTP ranged from 1-270 days, with a median of 21 days. Three individuals were frequent attenders to their GP and so we were unable to define the precise first date of presentation. Two thirds (68%) consulted their GP within 6 weeks of their trigger symptom. While more than half (58%) consulted their GPs only once before referral to secondary care, four patients (16%) saw their GP on more than three occasions.

For peer review only



For peer review only - http://bmjopen.bmj.com/site/about/guidelines.xhtml

**Table 1: Participant characteristics**

| | n=26 (%) | SYMPTOM Pancreas Study n= 391 (%) |
|---|---|---|
| **English region** | | |
| Eastern | 19 (73) | 372 (95) |
| North Eastern | 7 (27) | 19 (5) |
| **Gender** | | |
| Female | 14 (54) | 196 (50) |
| Male | 12 (46) | 195 (50) |
| **Age** | | |
| Mean Years (range) | 67 (48-84) | 69 (40-93) |
| 40-59 | 7 (27) | 89 (23) |
| 60-69 | 8 (31) | 111 (28) |
| 70-79 | 6 (23) | 127 (32) |
| 80+ | 5(19) | 64 (16) |
| **Ethnicity** | | |
| White British | 26 (100) | 384 (98) |
| **Educational qualifications** | | |
| Higher Education | 4 (15) | 145 (37) |
| Further Education/Secondary school | 14 (54) | 127 (33) |
| None | 8 (31) | 119 (30) |
| **IMD quintiles** | | |
| Least deprived 1 | 6 (23) | 125 (32) |
| 2 | 8 (31) | 133 (34) |
| 3 | 5 (19) | 71 (18) |
| 4 | 5 (19) | 43 (11) |
| Most deprived 5 | 2 (8) | 16 (4) |
| Missing | - | 3 (1) |
| **Diagnoses** | | |
| **Cancer** | | |
| Pancreas | 9 | 119 |
| Duodenal | 1 | 4 |
| Gallbladder | 1 | 11 |
| Oesophageal | 1 | 12 |
| Lymphoma | 1 | 7 |
| **Other conditions** | | |
| Hiatus Hernia | 3 | 28 |
| Gallstones | 2 | 25 |
| Pancreatitis | 2 | 9 |
| Others | 6 | 75 |
| **GP appointments made** | | |
| 0* | 2 (8) | |
| 1 | 15 (58) | |
| 2 | 5 (19) | |
| 3 or more | 4 (15) | |

*emergency presentation

## Qualitative themes

We were unable to find any clear differences regarding symptom appraisal or help-seeking between the accounts of people diagnosed with cancer and those with other conditions. We therefore report the findings together. We were also unable to find



For peer review only - http://bmjopen.bmj.com/site/about/guidelines.xhtml

For peer review only

any clear differences according to whether or not participants knew their diagnosis at the time of the interview, or their characteristics including gender, age and region. Extracts from interviews illustrate the findings: each quotation is contextualised, by the patient's gender (M/F), age (40-59, 60-69, 70-79, 80+), TTP (weeks), and diagnosis (cancer, non-cancer).

**1. Initial appraisal of symptoms**

Participants described how they first noticed and initially interpreted their symptoms and often attributed them to a common non-serious cause or condition.

***1.1 Bodily changes***

People mainly described initial subtle or non-specific bodily changes such as feeling tired or 'different'. These changes were often intermittent and insidious in their development.

*"I've had this sort of tiredness and a little bit of discomfort on and off for a time"* (M, 70-79, 13-26 weeks, cancer)

*"I've felt a bit like as if I've got a nervous tummy, like it gurgles up…"* (F 50-59, regular attender to GP, non-cancer)

People also described subtle changes in appetite: *"Well I went off my food a bit I think, my daughter said I went off my food a bit"* (F, 80+yrs, 2-4 weeks, cancer)

***1.2 Symptom attributions (Table 2)***

Initial explanations for these bodily changes or symptoms mainly related to diet and other daily activities. Nine participants with other conditions, particularly gastro-intestinal conditions such as gastro-oesophageal reflux disease, often appraised their new symptoms against their "usual" symptoms, and whether their treatment was still effective. This group tended to downplay their symptoms. Some people also attributed these bodily changes or symptoms to other medical conditions or side-effects of their medication. Others 'normalised' their initial symptoms to being caused by aging, common conditions such as a virus or common cold, indigestion or a urine infection.

These explanations were described in the rich context of people's lives, such as being busy, caring for others, or the time of the year. When considered in these contexts, most people initially felt that their symptoms were acceptable and did not warrant seeking help:

*"I thought I was a bit tired-er than normal, but I mean, I was tearing around. My daughter was coming back for three months and they've got a house in the next village, I was over there doing things in the house, getting it ready."* (F, 60-69, 2-4 weeks, cancer)

For peer review only

**Table 2: Illustrative quotations relating to initial explanations**

| | Illustrative quotations |
|---|---|
| Diet and lifestyle | *"It comes and goes, it must be something that I eat and it just doesn't agree with me"* (F, 60-69, 13-26 weeks, non-cancer)<br><br>*"I think it's the picking up, it's the bending that does it a lot with my job as well, 'cos I lift heavy (clothing) all day, and I'm putting them over people and then bending and sitting down"* (F, 40-59, regular attender to GP, cancer) |
| Existing gastrointestinal conditions | *"I've always suffered with what's it called, you know, reflux, over the years, sometimes I could take some indigestion remedy and that Gaviscon thing and it'd calm down"* (F, 40-49, <2 weeks, non-cancer) |
| Other medical conditions, side-effects of medication | *"At first I started putting it down to the diabetic pills, I thought 'Well it's probably because I've changed those', you know, and this Pioglitazone, I kept thinking 'Well it's that',* (F 60-69, 2-4 weeks, cancer)<br><br>*"I've had this sort of tiredness and a little bit of discomfort on and off for a time, I thought it was possibly diabetes…it kept on… and we thought was perhaps as a result of some of the medication I was taking for other things."* (M, 70-79, 13-26 weeks, cancer) |

***2. Responses to initial symptom appraisal***

***2.1 Lifestyle adaptations***

In the light of these attributions and explanations, some people began to make adaptations to their diet or daily living activities to alleviate their symptoms; in some cases, these changes were quite significant:

*"If I had smaller meals, but slightly more frequently, I could actually control keeping food, you know, keeping comfortable"* (M, 60-69, 5-8 weeks, cancer)

*"I think you learn to live with it and you just adapt to it and start to make the changes so it doesn't happen…. I think what had happened with me is I couldn't control it any longer, no matter if I stopped eating things, didn't eat out, it was happening anyway"* (F, 60-69, 26-52 weeks, non-cancer)

***2.2 Role of others in appraisal and adaptations***

Some people discussed their early symptoms with close relatives, particularly partners or children. This could prompt discussions around potential explanations and suggestions for improving symptoms, such as changes in content or timing of meals; at times it could also lead to delays in helpseeking:

*"When I mentioned to my wife the colour of my stools, which was the first symptom, she said I wasn't getting enough carbohydrates or protein or something…something wasn't right in my diet. So I changed my diet, I was eating fruit and vegetables and things like that, a lot more, and it still stayed the same."* (M, 50-59, 13-26 weeks, cancer)



For peer review only - http://bmjopen.bmj.com/site/about/guidelines.xhtml

**3. Further appraisal of symptoms**

Changes in patterns of symptoms often led patients to further appraise their symptoms, and to question the reasons for their occurrence and their original explanations.

***3.1 Symptom progression (Table 3)***

Symptoms became of greater concern when they became more severe or frequent, or when further subtle symptoms developed. In some cases, it appeared that there was progression from subtle and intermittent symptoms of a manageable and tolerable nature, to symptoms which were more alarming such as pain. Across the whole sample, people described pain in a number of different parts of the body including their shoulder, chest, upper abdomen and back.

Another man described the chronology of his symptoms; having started with mild indigestion symptoms, additional, more alarming, symptoms developed: *"gradually… the stools over a period of time, but the urine it seemed to happen more or less in a week, from normal colour to really deep colour"* (M, 50-59, 13-26 weeks, cancer)

Many participants reported subtle yet progressive changes to their appetite and enjoyment of food. Weight loss as a result of changes in appetite was also reported by a few participants.

***3.2 Symptom monitoring and self-management***

The subtlety and slow progression of symptoms prompted some participants to monitor their symptoms, and some to consider alternative self-management strategies. Many used over-the-counter remedies to attempt to alleviate their symptoms of indigestion or abdominal pain:

*"It was only when I started feeling a bit of strangeness that I decided to try and keep a pattern of them"* (F, 60-69, 2-4 weeks, cancer)

***3.3 Impact on life***

Most people described how their symptoms gradually or rapidly impacted on their quality of life and daily routine, including their social activities and hobbies, as well as their ability to sleep. Some felt that they were less able to complete tasks at home; for others, work became more challenging:

*"I decided I'd got this burning pain and I noticed that I was beginning to get a little bit short of breath, and also I couldn't do the same things I used to, like I found digging was becoming impossible"* (M, 70-79, 13-26 weeks, non-cancer)

***3.4 Previous family and personal history of cancer***

A few people expressed a concern that they might develop cancer because they had lost a close relative to the disease; one had also previously suffered from cancer. These people were concerned about their personal risk of cancer and reported that they often considered whether their symptoms matched those which they expected from having cancer. Conversely one woman, subsequently diagnosed with a benign condition, had not considered cancer as a possibility because her symptoms did not match her expectations of cancer:



For peer review only - http://bmjopen.bmj.com/site/about/guidelines.xhtml

For peer review only

*"I knew I'm generally healthy, I mean I've been going swimming and to the gym…it wasn't seriously debilitating…I expect when you have cancer that you have more symptoms"* (F, 60-69, 26-52 weeks, non-cancer)

**Table 3: Illustrative quotations relating to further appraisal of symptom progression**

| | Illustrative quotations |
|---|---|
| Pain | *"Well, it varied, you know, sometimes I'd have the pain all day and that's very wearing and painful…It's just under the rib cage and it's slightly to the right hand side, just slightly there, always in the same place."* (F, 70-79, <2 weeks, non-cancer)<br><br>*"…I just had a nagging, more like a dull ache, underneath my bust and I thought it was indigestion and it didn't seem to go away, it didn't make any difference whether I ate or not, it was there quite a lot of the time"* (F, 70-79, 2-4 weeks, cancer)<br><br>*"After Christmas that then became all day, every day. I was going to bed with a stomach ache and I was waking up with a stomach ache…It was there all the while and you know, it didn't make sense that I'd have a problem all the while so I thought I was imagining it"* (M, 60-69, 2-4 weeks, cancer) |
| Progressive changes in appetite and enjoyment of food | *"I was actually going off the actual taste of food, I wasn't enjoying food anymore, things that I would normally eat I wasn't enjoying"* (M, 60-69, 5-8 weeks, cancer)<br><br>*"Well it became quite traumatic at one point, when I just [did] not want an evening meal at all. I always eat a reasonable breakfast, but for the evening meal I had no appetite"* (M, 80+, <2 weeks, cancer)<br><br>*… and I was eating less and smaller meals and being really careful what I ate, and I thought "it's good for you but you shouldn't have to be that precise when you eat"* (F, 60-69, 26-52 weeks, non-cancer) |
| Weight loss | *"I was also suffering uncomfortable, you know, let's say stomach irritation, indigestion, wasn't happy eating, so I literally went along to say 'look, this isn't right, I don't mind losing weight but not like this"* (M 60-69, 5-8 weeks, cancer)<br><br>*"I'd probably lost about four pounds which was concerning me but then I thought, you've reduced the amount that you're eating…"* (F 50-59, 2-4 weeks, non-cancer) |

**4. Deciding to seek help (Table 4)**

The further appraisal of symptoms eventually led most participants to decide to seek help from primary care, although several had emergency visits to A&E.

***4.1 Triggers***



For peer review only - http://bmjopen.bmj.com/site/about/guidelines.xhtml

Triggers to seek help usually involved an increased frequency or intensity of symptoms, although sometimes it was precipitated by an alarming event, usually pain. Pain was often the "tipping point" to seek help against a background of other more subtle symptoms. For those without pain, triggers were often complex, and could involve interpreting new or changing symptoms alongside symptoms from other existing conditions and, in some instances, symptoms from other acute illnesses. Healthcare factors, such as availability of a GP appointment, were seldom mentioned.

***4.2 Legitimising help-seeking***

People were often encouraged to seek help by their spouse, family members and friends; they were particularly influenced by comments about changes in their general wellbeing, or recall of specific symptom episodes. A few participants reported considering whether their symptoms had become a 'legitimate' reason to seek healthcare when self-management strategies became ineffective; they tended to refer to the length of time symptoms had been experienced, and their reduced ability to cope.

**Table 4: Illustrative quotations relating to deciding to seek help**

| | Illustrative quotations |
|---|---|
| Pain on a background of more subtle symptoms | *"Sometimes I could take some indigestion remedy and.. it'd calm down, but this were a different kind of pain, this were, oh it's excruciating pain…but that were the first time that… I thought there's something not right here and I need to get some help."* (F, 40-49, <2 weeks, non-cancer) |
| Help-seeking encouraged by others | *"The kids had been saying "mum you're always ill" and I thought "oh yeah, I am". Because you, I think you sort of drift, you get used to things and you don't see any problem with it, and when other people start to say, you know "hang on" and then I thought "oh yeah"*(F, 60-69, 26-52 weeks, non-cancer)<br><br>*"I'd fall asleep on my desk, you know, this isn't me, and other people said "oh you're losing weight"… people actually were saying "you don't look well". It's another lever to go and see, to get it sorted"* (M, 60-69, 5-8 weeks, cancer) |
| Legitimate reason | *"I think my basic problem was pain… from arthritis that I'd had for some time, but it was proving impossible to control it by the methods I was using"* (M 80-89, 2-4 weeks, cancer) |

**DISCUSSION**



For peer review only - http://bmjopen.bmj.com/site/about/guidelines.xhtml

This is the first study to investigate symptom appraisal and help-seeking in patients newly referred to hospital with symptoms suggestive of pancreatic cancer. Due to their subtle and intermittent pattern, many people initially just monitored their symptoms. Symptoms were interpreted based on previous experiences with common conditions such as gastroenteritis, or impact of dietary change or existing gastro-intestinal conditions. Many made changes to their eating patterns including type of food, and frequency and quantity of meals, or managed their symptoms with over-the-counter medication. Consequently, they tended not to go to their GP at that point. Changes to symptom frequency, duration or severity, their impact on daily life, or the appearance of additional symptoms, particularly pain, led many people to further appraise their symptoms and visit their GP. Family and friends often made important contributions to encouraging help-seeking by reflecting on changes in symptoms, although at times social contacts could re-inforce self-management strategies. These patterns of symptom appraisal and help-seeking were seen regardless of final diagnosis.

**Strengths and limitations**

The major strength of this study is that we prospectively recruited patients with symptoms suggestive of pancreatic cancer, and interviewed them during their pathway to diagnosis and treatment. This allowed detailed discussions about factors affecting symptom appraisal and help-seeking while they were still fresh events, and greatly reduced the possibility of *post-hoc* rationalisation and recall bias. We have used similar approaches in interview studies with people recently diagnosed with melanoma [22], and people with symptoms suggestive of lung [10] and colorectal [12] cancers. Recruiting patients during their diagnostic pathway enabled comparisons about symptom appraisal and help-seeking between people subsequently diagnosed with cancer and people with non-cancer conditions; importantly, we were not able to find differences between them. Our recruitment process meant that some patients were subsequently diagnosed with a cancer other than pancreatic cancer. We do not consider this a weakness of the study; rather, it serves to highlight the complexity and challenges of diagnosing pancreatic cancer, and how similarly a range of upper gastro-intestinal cancers can present. While we aimed to recruit equal numbers of patients with pancreatic cancer and benign conditions, several patients with cancer withdrew before interview as they were too unwell.

Our approach to data collection and analysis was strengthened by the use of a strong theoretical framework: underpinned by recommendations from the Aarhus statement on improving design and reporting studies on early cancer diagnosis [18], we applied the framework of the widely used Model of Pathways to Treatment [16,17], and a specifically-developed calendar-landmarking instrument which aided



For peer review only - http://bmjopen.bmj.com/site/about/guidelines.xhtml

some participants' recall of dates of their symptoms and help-seeking [14]. Other strengths include recruitment from two English regions and a number of hospitals to ensure patients from a wide range of socio-economic backgrounds, and who had entered hospital care via a variety of routes, enabling us to gather a broad range of experiences. Finally, the research team had a broad range of scientific and clinical expertise, and we sought input from our lay members at all stages of the research process, including analysis and interpretation of the data.

While we acknowledge that these experiences may not be representative of all people with symptoms suggestive of pancreatic cancer, the demographics of this cohort are similar to that of the main SYMPTOM-Pancreas study. Although a major finding was that there were similar patterns in symptom appraisal and help-seeking between those diagnosed with cancer and those with other diagnoses, this may have been partly due to recruiting patients relatively late in their diagnostic pathway, when GPs had referred with a suspicion of pancreatic cancer. Ideally we would have recruited people with similar symptoms from earlier in their diagnostic pathway and from primary care, but there are significant challenges with the feasibility and costs of recruiting such a cohort containing sufficient cases of pancreatic cancer. Finally, we can only report the experiences shared with the researchers at the time of the interview: there may have been events which were not discussed if they were felt to be too emotional or private.

**Comparison with existing literature**

Although these findings are in line with the broader literature investigating help-seeking for cancer symptoms [10,12, 22], very little has been published on symptom appraisal and help-seeking among patients diagnosed with pancreatic cancer. Evans *et al.* recently reported the only other similar study, also set in England, and highlighted the intermittent and subtle nature of symptoms with their title: '"It can't be very important because it comes and goes"- patients' accounts of intermittent symptoms preceding a pancreatic cancer diagnosis' [8]. However, their interviews were conducted with people up to several years after a diagnosis of pancreatic cancer, and included data from interviews with relatives of people who had died. Our findings are strengthened by interviewing patients earlier in their illness pathway, thereby capturing subtle assessments of initial bodily changes and explanations, and minimising post-hoc rationalisation and recall bias. We could not identify any subtle differences in the nature of symptoms or help-seeking between those who eventually were found to have pancreatic cancer, and those who were not. This is consistent with the quantitative findings of the SYMPTOM-pancreas study [9], where the evolution of additional symptoms such as fatigue, change in bowel habit, weight loss, decreased appetite and jaundice were associated with pancreatic cancer.



For peer review only - http://bmjopen.bmj.com/site/about/guidelines.xhtml

Other studies of pancreatic cancer symptoms have mainly used quantitative retrospective designs. For example, Stapley *et al*. undertook a case-control study using routinely recorded UK primary care data which demonstrated how poorly predictive most symptoms are of pancreatic cancer in primary care populations. While the positive predictive value of jaundice was relatively high (21%), it was much lower for other symptoms including abdominal and back pain, nausea, vomiting, change in bowel habit, weight loss and malaise (<1%) [6]. Keane *et al*. more recently used another large UK primary care database to investigate the early symptom profiles of patients diagnosed with pancreatic ductal or biliary adenocarcinoma and found a similar wide range of early symptoms [23]. They also found that, in the year prior to diagnosis, these patients visited their GP on a median of 18 (IQR 11–27) occasions, which resonated with findings from another large English database study showing that 41% of patients diagnosed with pancreatic cancer had visited their GP at least three times before referral to specialist care [7]. In contrast, more than half our sample reported only visiting their GP once before referral, suggesting either that our sample were recruited early in their symptom pathway, or perhaps that they had symptoms such as jaundice which are more likely to prompt early referral. Other quantitative studies set among patients newly diagnosed with pancreatic cancer patients have reported fatigue, dyspepsia and subtle changes in appetite such as early satiety in the preceding six months, confirming our finding that there may be a window of opportunity for timely investigations when patients report combinations of non-specific or subtle symptoms [4,24,25].

**Implications for research and practice**

Our findings add to current knowledge about the subtle and non-specific nature of early symptoms of pancreatic cancer, and enhance our understanding of their often intermittent and insidious nature. They highlight the importance of on-going, and often iterative, appraisal of symptoms as described in the Model of Pathways to Treatment [16,17]. The findings have characterised pain as a 'tipping point' for presentation to healthcare, and underpinned the importance of family and friends in symptom monitoring, recognising evolving and new symptoms and endorsement of help-seeking [26]. The challenge for symptom awareness campaigns is to promote earlier presentation with these more subtle and intermittent symptoms which lead to changes in eating patterns including type of food, and frequency and quantity of meals, and weight loss. The 'Know 4 Sure' campaign was one of the first to raise awareness of non-site-specific symptoms including weight loss, pain, lump and unexplained bleeding. It was associated with increase in referrals for a range of cancers including upper GI cancer [27]. Whether public health campaigns can effectively promote messages about combinations of non-specific symptoms is unknown, but may be important in pancreatic cancer.



For peer review only - http://bmjopen.bmj.com/site/about/guidelines.xhtml

The newly revised NICE guidance on Suspected Cancer: Recognition and Referral [28] supports a lower threshold for investigating and referring people with single and multiple symptoms suggestive of pancreatic cancer, particularly those in higher risk groups such as newly diagnosed diabetics. The findings from this study could inform GP education approaches to promote timely investigation and referral for people with these non-specific and intermittent symptoms. These numerous subtle and non-specific symptoms, when combined with abnormal blood tests and the use of clinical decision support tools, might alert GPs to a possible pancreatic cancer diagnosis earlier. Appropriate safety-netting is also important due to the evolving nature of symptoms, including symptom monitoring and early review.

In conclusion, this study has described and contextualised the appraisal and help-seeking behaviours of patients with symptoms that could represent pancreatic cancer. Greater awareness of combinations of the subtle and intermittent symptoms, and their evolving nature, is needed to prompt timelier help-seeking and investigation among people with symptoms of pancreatic cancer.

For peer review only



For peer review only - http://bmjopen.bmj.com/site/about/guidelines.xhtml

**Acknowledgments**

We thank the patients who so kindly and freely gave their time and experiences to this study. We thank Helen Morris the SYMPTOM study manager, our other study patient and public representatives Sue Ballard and Victor Boulter, and the staff at the participating hospitals (Bedford Hospital NHS Trust, Cambridge University Hospitals NHS Foundation Trust; Hinchingbooke Healthcare NHS Trust, The Queen Elizabeth Hospital Kings Lynn NHS Trust, Peterborough and Stamford NHS Foundation Trust, University Hospital of North Durham; Darlington Memorial Hospital: University Hospital of North Tees, West Suffolk NHS Foundation Trust) who facilitated participant recruitment and supported the study. The authors would also like to acknowledge the contribution made by the NIHR funded Discovery Programme Steering Committee comprising: Roger Jones (chair); Alison Clutterbuck; Ardiana Gjini; Joanne Hartland; Maire Justice; Jenny Knowles; Richard Neal; Peter Rose.

**Contributors**

FMW, JE, GR and WH were responsible for study design, study conduct and management. KM, LB and NH collected the data, and with the support of JB, JE, JL, MJ and FMW, KM analysed and interpreted the data. KM led on the manuscript preparation. All authors contributed to the writing and completion of the paper and have approved the final the manuscript.

**Funding**

This paper presents independent research funded by the National Institute for Health Research Programme Grants for Applied Research programme (RP-PG-0608-10045) and Pancreatic Cancer Action (registered charity 1137689). The views expressed are those of the authors and not necessarily those of the NHS, the NIHR or the Department of Health. The funders of this study had no role in study design, data collection, analysis, and interpretation, or the writing of this research paper. FMW is supported by an NIHR Clinician Scientist award.

**Competing interests**

The authors have no conflicts of interest to declare.

**Data sharing statement**

No additional data are available.

**Ethics approval**

Ethical approval was granted by the Cambridgeshire 3 Research Ethics Committee (10/H0306/50).

For peer review only

**REFERENCES**

1. Cancer Research UK, *Incidence Statistics* 2011. Retrieved from http://www.cancerresearchuk.org/health-professional/cancer-statistics/incidence/common-cancers-compared#heading-Zero (Last accessed 31.05.16).

2. Hiom S. Diagnosing cancer earlier: reviewing the evidence for improving cancer survival. *Br J Cancer* 2015; 112 (supp1): S1-S5.

3. Neal RD, Tharmanathan P, France B et al. Is increased time to diagnosis and treatment in symptomatic cancer associated with poorer outcomes? Systematic review. Br J Cancer 2015; 112:S92-S107.

4. Holly EA, Chaliha I, Bracci PM, et al. Signs and symptoms of pancreatic cancer: a population-based case-control study in the San Francisco Bay area. *Clin Gastroenterol Hepatol* 2004; 2:510-7.

5. Hippisley-Cox J, Coupland C. Identifying patients with suspected pancreatic cancer in primary care: derivation and validation of an algorithm. *Br J Gen Pract* 2012; 62: e38-45.

6. Stapley S, Peters TJ, Neal RD, et al. The risk of pancreatic cancer in symptomatic patients in primary care: a large case-control study using electronic records. *Br J Cancer* 2012; 106: 1940-4.

7. Lyratzopoulos G, Neal RD, Barbiere JM, et al. Variation in number of general practitioner consultations before hospital referral for cancer: findings from the 2010 National Cancer Patient Experience Survey in England, *The Lancet Oncology* 2012; 13: 353–365.

8. Evans J, Chapple A, Salisbury H, et al. "It can't be very important because it comes and goes"--patients' accounts of intermittent symptoms preceding a pancreatic cancer diagnosis: a qualitative study. *BMJ Open* 2014; 4: e004215.

9. Walter FM, Mills K, Mendonca SC, et al. Symptoms and patient factors associated with diagnostic intervals for pancreatic cancer (SYMPTOM pancreatic study): a prospective cohort study. Lancet Gastroenterol Hepatol 2016; 1:298-306.

10. Birt L, Hall N, Emery J, et al. Responding to symptoms suggestive of lung cancer: a qualitative interview study. *BMJ Open Respir Res* 2014; 1(1): e000067.

11. Walter FM, Rubin G, Bankhead C, et al. Symptoms and other factors associated with time to diagnosis and stage of lung cancer: a prospective cohort study. *Br J Cancer* 2015; 112 (supp1): S6-S13.

12. Hall N, Birt L, Banks J et al. Symptom appraisal and healthcare-seeking for symptoms suggestive of colorectal cancer: a qualitative study. *BMJ Open* 2015;5(10):e008448.



For peer review only - http://bmjopen.bmj.com/site/about/guidelines.xhtml

13. Emery JD, Walter FM, Gray V, et al. Diagnosing cancer in the bush: a mixed methods study of GP and specialist diagnostic intervals in rural Western Australia. *Fam Pract* 2013; 30(5):541-50.

14. Mills K, Emery J, Cheung C, et al. A qualitative exploration of the use of calendar landmarking instruments in cancer symptom research. BMC *Fam Pract* 2014; 15:167.

15. Guest G, Bunce A, Johnson L. How many interviews are enough? An experiment in data saturation and variability. *Field Methods* 2006; 18:59-82.

16. Walter FM, Scott SE, Webster A, et al. The Andersen Model of Total Patient Delay: a systematic review of its application in cancer diagnosis. *J Health Services Research & Policy* 2012; 17(2):110-8.

17. Scott SE, Walter FM, Webster A et al. The model of pathways to treatment: conceptualization and integration with existing theory. *Br J Health Psychol* 2013; 18(1): 45-65.

18. Weller D, Vedsted P, Rubin G, et al. The Aarhus statement: improving design and reporting of studies on early cancer diagnosis. *Br J Cancer* 2012; 106(7):1262-1267.

19. Braun, V. and Clarke, V. Using thematic analysis in psychology. Qualitative Research in Psychology, 2006, 3 (2). pp. 77-101.

20. Gale NK, Heath G, Cameron E, Rashid S, Redwood S. Using the framework method for the analysis of qualitative data in multi-disciplinary health research, BMC Med Res Methodol. 2013 Sep 18;13:117.

21. Ritchie J, Spencer L. Qualitative data analysis for applied policy research. In: Bryman A, Burgess R, eds. *Analysing qualitative data*. London: Routledge, 1993:173-94.

22. Walter FM, Birt L, Cavers D, et al. This isn't what mine looked like: a qualitative study of symptom appraisal and help-seeking in people recently diagnosed with melanoma. *BMJ Open* 2014; 21: 4(7) e005566.

23. Keane MG, Horsfall L, Rait G, Pereira SP. A case-control study comparing the incidence of early symptoms in pancreatic and biliary tract cancer. *BMJ Open* 4:e005720.

24. Gullo L, Tomassetti P, Migliori M, et al. Do early symptoms of pancreatic cancer exist that can allow an earlier diagnosis? *Pancreas* 2001; 22:210-3.

25. Ingeman ML, Christensen MB, Bro F. The Danish cancer pathway for patients with serious non-specific symptoms and signs of cancer–a cross-sectional study of patient characteristics and cancer probability, *BMC Cancer* 2015; 15:421.

For peer review only

26. Leydon GM, Bynoe-Sutherland J, Coleman MP. The journey towards a cancer diagnosis: the experiences of people with cancer, their family and carers. *Eur J Cancer Care* 2003; 12:317-326.

27. Cancer Research UK, "Know 4 sure" campaign 2014. Retrieved from http://www.cancerresearchuk.org/health-professional/early-diagnosis-activities/be-clear-on-cancer/know-4-sure-campaign (Last accessed 31.05.2016).

28. National Institute for Health and Care Excellence, Suspected Cancer: Recognition and Referral 2015. Retrieved from http://www.nice.org.uk/guidance/ng12 (Last accessed 31.05.16).

For peer review only

BMJ Open

# Understanding symptom appraisal and help-seeking in people with symptoms suggestive of pancreatic cancer: a qualitative study.

| | |
|---|---|
| Journal: | *BMJ Open* |
| Manuscript ID | bmjopen-2016-015682.R2 |
| Article Type: | Research |
| Date Submitted by the Author: | 07-Jul-2017 |
| Complete List of Authors: | Mills, Katie; University of Cambridge, Public Health & Primary Care<br>Birt, Linda; University of East Anglia, School of Health Sciences<br>Emery, Jon; University of Melbourne, General Practice and Primary Care Academic Centre<br>Hall, Nicola; Durham University School of Medicine Pharmacy and Health<br>Banks, Jonathan; University of Bristol, Social & Community Medicine<br>Johnson, Margaret ; University of Cambridge, Patient Representative c/o Public Health & Primary Care<br>Lancaster, John; University of Cambridge, Patient Representative c/o Public Health & Primary Care<br>Hamilton, Willie; University of Exeter Medical School, Primary Care Diagnostics<br>Rubin, Greg; Durham University, School of Medicine and Health<br>Walter, Fiona; University of Cambridge, Dept of Public Health and Primary Care |
| <b>Primary Subject Heading</b>: | Qualitative research |
| Secondary Subject Heading: | General practice / Family practice, Gastroenterology and hepatology |
| Keywords: | pancreatic cancer, diagnosis, appraisal, help-seeking, symptoms |
| | |

SCHOLARONE™
Manuscripts

# Understanding symptom appraisal and help-seeking in people with symptoms suggestive of pancreatic cancer: a qualitative study.

**Author names, affiliations, full address and postcode, contact details:**

**Katie Mills (Corresponding author**- ko298@medschl.cam.ac.uk)
Research Associate
The Primary Care Unit
Department of Public Health and Primary Care
University of Cambridge
Cambridge CB1 8RN

**Linda Birt** Linda.Birt@uea.ac.uk
At the time of completing this research:
Research Associate
The Primary Care Unit
Department of Public Health and Primary Care
University of Cambridge
Cambridge CB1 8RN

**Jon D Emery** jon.emery@unimelb.edu.au
Herman Professor of Primary Care Cancer Research
Department of General Practice
University of Melbourne
Australia

**Nicola Hall** n.hall@sunderland.ac.uk
At the time of completing this research:
Research Associate
School of Medicine, Pharmacy & Health
Durham University
Stockton on Tees TS17 6BH

**Jonathan Banks** Jon.Banks@bristol.ac.uk
Research Fellow
School of Social and Community Medicine
University of Bristol
Bristol BS8 2PS

**Margaret Johnson**
Patient representative c/o: ottersriverbank@natsirt.co.uk
Primary Care Unit
Department of Public Health & Primary Care



For peer review only - http://bmjopen.bmj.com/site/about/guidelines.xhtml

University of Cambridge
Cambridge CB1 8RN

**John Lancaster** lancaster366@btinternet.com
Patient representative c/o:
Primary Care Unit
Department of Public Health & Primary Care
University of Cambridge
Cambridge CB1 8RN

**William Hamilton** W.Hamilton@exeter.ac.uk
Professor of Primary Care Diagnostics
College House, St Luke's Campus
University of Exeter
Exeter EX2 4TE

**Greg P Rubin** g.p.rubin@durham.ac.uk
Professor of General Practice and Primary Care
School of Medicine, Pharmacy & Health
Durham University
Stockton on Tees TS17 6BH

**Fiona M Walter** fmw22@medschl.cam.ac.uk
Principal Researcher in Primary Care Cancer Research
Primary Care Unit
Department of Public Health & Primary Care
University of Cambridge
Cambridge CB1 8RN

**Keywords**: pancreatic cancer, diagnosis, appraisal, help-seeking, symptoms



For peer review only - http://bmjopen.bmj.com/site/about/guidelines.xhtml

## ABSTRACT

### Objective

Pancreatic cancer has poor survival rates due to non-specific symptoms leading to later diagnosis. Understanding how patients interpret their symptoms could inform approaches to earlier diagnosis. This study sought to explore symptom appraisal and help-seeking among patients referred to secondary care for symptoms suggestive of pancreatic cancer.

### Design

Qualitative analysis of semi-structured in-depth interviews. Data were analysed iteratively and thematically, informed by the Model of Pathways to Treatment.

### Participants and setting

Pancreatic cancer occurs rarely in younger adults, therefore patients aged ≥ 40 years were recruited from nine hospitals after being referred to hospital with symptoms suggestive of pancreatic cancer; all were participants in a cohort study. Interviews were conducted soon after referral, and where possible, before diagnosis.

### Results

Twenty-six interviews were conducted (cancer n=13 (pancreas n=9, other intra-abdominal n=4), non-cancer conditions n=13; age range 48-84 years; 14 women). Time from first symptoms to first presentation to healthcare ranged from 1 to 270 days, median 21 days. We identified three main themes. Initial symptom appraisal usually began with intermittent, non-specific symptoms such as tiredness or appetite changes, attributed to diet and lifestyle, existing gastrointestinal conditions or side-effects of medication. Responses to initial symptom appraisal included changes in meal type or frequency, or self-medication. Symptom changes such as alterations in appetite and enjoyment of food or weight loss usually prompted further appraisal. Triggers to seek help included a change or worsening of symptoms, particularly pain, which was often a 'tipping point'. Help-seeking was often encouraged by others. We found no differences in symptom appraisal and help seeking between people diagnosed with cancer and those with other conditions.

### Conclusions

Greater public and healthcare professional awareness of the combinations of subtle and intermittent symptoms, and their evolving nature, is needed to prompt timelier help-seeking and investigation among people with symptoms of pancreatic cancer.


For peer review only



For peer review only - http://bmjopen.bmj.com/site/about/guidelines.xhtml

**STRENGTHS AND LIMITATIONS OF THIS STUDY**

- We believe this is the first study to compare the symptom appraisal and help-seeking experiences of patients referred to secondary care with symptoms suggestive of pancreatic cancer between people subsequently diagnosed with pancreatic cancer and people diagnosed with other non-cancer conditions. We were unable to identify any differences.

- The data collection and analysis of this study were guided by the model of pathways to treatment as recommended by the Aarhus Statement to improve the design and reporting of studies on early cancer diagnosis.

- Risks of recall bias and post hoc rationalisation were reduced by recruiting at the time of referral to specialist care, and interviewing patients before or close to diagnosis.

- The subtlety of initial symptoms and their evolution over time is a novel finding, and provides some understanding of the complexities faced by patients when appraising their symptoms and seeking help in a timely way.

- Some of the most seriously ill patients were unable to be interviewed and their experiences may have differed from those in our sample.

For peer review only

**INTRODUCTION**

Pancreatic cancer is the ninth most common cancer in the UK, with about 8,000 cases diagnosed every year. Only 22% men and 20% women currently survive pancreatic cancer for one year or more, and it has the poorest 5-year survival rates of all cancers at less than 4% [1]. This poor prognosis is mainly due to patients being diagnosed at a stage when curative treatment is not possible. Survival could be improved if patients could be diagnosed earlier [2,3].

The key symptoms of pancreatic cancer have primarily been described from studies of patients after a diagnosis has been made. Pre-diagnosis case-control studies from the US [4] and UK [5,6] confirm that these symptoms are also relevant for the community when making healthcare management decisions. While jaundice is the only symptom which is strongly predictive of pancreatic cancer, a number of non-specific symptoms such as nausea, vomiting, abdominal pain, back pain, constipation, diarrhoea, weight loss and malaise are also weakly associated [6]. The non-specific nature of these symptoms creates challenges for early recognition of a potentially serious condition by both patients and their GPs. In a large English database study, over 40% of patients diagnosed with pancreatic cancer had visited their GP at least three times before referral to specialist care [7].

There is limited evidence about the evolution of symptoms of pancreatic cancer over time, and how patients appraise their symptoms and decide to seek medical advice. One of the few studies to date interviewed patients or their relatives many months after diagnosis [8]; such studies may be biased by *post hoc* rationalisation and recall bias. Furthermore, many symptoms of pancreatic cancer are also symptoms of other gastrointestinal or intra-abdominal cancers such as gallbladder, colon and ovarian cancer, which may be equally difficult to diagnose early [7].

The aim of this study was therefore to use qualitative approaches to gain understanding of barriers and facilitators to symptom appraisal and help-seeking decisions among patients with symptoms suggestive of pancreatic cancer much earlier in their diagnostic pathway, to contribute to the development of interventions to promote earlier or more timely cancer diagnosis. Similar studies have been conducted for people with symptoms suggestive of lung and colorectal cancer [9-11]. We recruited patients who were newly referred to hospital, thus providing the opportunity to investigate the complexities of patients' symptom appraisal and decision making, and explore pathways from their first symptom to first presentation in primary care, emergency presentations, and referrals to specialist services.



For peer review only - http://bmjopen.bmj.com/site/about/guidelines.xhtml

**METHODS**

**Setting**

This in-depth, face-to-face interview study was nested within the SYMPTOM Pancreas Study, a prospective cohort study investigating associations between symptoms and other factors on the total diagnostic interval and stage of diagnosis among patients with symptoms suggestive of pancreatic cancer [12]. The patients were recruited from nine hospitals in two regions of England (North East and Eastern). Ethical approval for all the SYMPTOM studies was obtained from Cambridgeshire 3 Research Ethics Committee (10/H0303/50).

**Recruitment**

Patients were recruited when referred to hospital via routine or urgent (two-week wait, '2WW') routes to a gastroenterology or hepato-pancreato-biliary (HPB) clinic or relevant ultrasound department, or admitted via A&E or other specialists. Pancreatic cancer occurs rarely in younger adults, therefore patients were eligible if they were aged 40 years and over, and their GPs had reported symptoms suggestive of pancreatic cancer. They were initially sent an invitation letter and SYMPTOM study questionnaire by post; people who completed the questionnaire were able to indicate whether they would be willing to take part in an interview. We purposively sampled patients (by region, age, gender, diagnosis) to obtain participants with a range of demographic and diagnosis characteristics.

**Data collection**

Interviews were undertaken between March 2012 and March 2014 by KM, LB and NH, all of who have extensive qualitative research experience. They took place as soon as possible after referral to hospital. All diagnoses were confirmed from review of secondary care medical records.

Before starting the interview, the researcher explained the research process and written consent was obtained from the participant. The interview was semi-structured, with the schedule (supplementary file 1) developed from similar interviews undertaken with people recently diagnosed with cancer [13]. By using open-ended questions we were able to explore the patient's appraisal of their symptoms and decisions to seek help. We encouraged the interviewees to share how they made sense of their symptoms and why they chose to seek help in the context of previous experiences and existing medical conditions. To assist in the clarification of the sequences of events and key dates along the pathway to diagnosis, we used a specifically designed calendar-landmarking instrument [14].

The interviews lasted between 40-70 minutes and were conducted in participants' homes. A relative, usually a spouse, was also present at several interviews at the



For peer review only - http://bmjopen.bmj.com/site/about/guidelines.xhtml

participant's request: they sometimes assisted in recall of events and confirmed participants' accounts, but were not consented, and only the participant's words were included as data in analyses. Interviews continued until no new themes were identified in three consecutive interviews and data saturation was reached [15]. All interviews were audio-recorded, then professionally transcribed verbatim and anonymised via a confidential service.

**Analyses**

***Theoretical model***

Both data collection and analysis were underpinned by the theoretical approach of the Model of Pathways to Treatment [16,17] as recommended by the Aarhus Statement to improve the design and reporting of studies on early cancer diagnosis [18]. The Model considers the contributions of disease, patient and healthcare system factors to four intervals: Appraisal, Help-seeking, Diagnostic and Pre-Treatment. The first event is the detection of initial bodily changes, described as the "time point when a person becomes aware of body sensations or visual alterations, regardless of the meaning assigned to the change", these then develop over time to "symptoms", defined as a bodily change perceived to be abnormal. Symptom appraisal leads to perceiving a reason to seek help, and consulting with a healthcare professional. These key events represent the "time to presentation" (TTP) period. We defined TTP as the interval between the patient-reported date of first noticing a symptom and their first consultation with a healthcare professional.

***Analytic processes***

The analytic process was iterative, starting after the first few interviews. Three researchers with social sciences expertise (KM, LB, NH) worked together on an initial thematic analysis of inductive coding [19], and then adopted a deductive approach guided by the framework of the Model of Pathways to Treatment [16,17,20] to construct themes and sub-themes. These approaches were combined to ensure a rigorous and systematic progression through the five analytic steps: familiarisation with data, developing a thematic framework; indexing data to framework; mapping and questioning the data; and theoretical interpretation [21]. The Model was used at several points in the analysis process: first, to provide structure to understanding the patient pathways to diagnosis; second, to facilitate comparisons between participants diagnosed with cancer and those who presented with similar symptoms but were diagnosed with non-cancer conditions. Finally, the researchers examined patient data by characteristics, including age, gender, co-morbidity, geographical region and diagnosis, to seek patterns and non-confirming cases.

The study patient representatives (MJ, JL) have personal experiences of cancer, which was reflected in their interpretation of the data. They contributed to all stages



For peer review only - http://bmjopen.bmj.com/site/about/guidelines.xhtml

of analysis by reading transcripts and regular meetings with the research team, and the other authors (all with clinical or social science expertise) contributed to the interpretation of the data. Data management was assisted by NVivo version 9.

## RESULTS

### Sample (Table 1)

302/407 (74%) of the participants recruited into the main SYMPTOM Pancreas Study expressed an interest in an interview. Thirty-four individuals were recruited following purposive sampling, but 8 were not interviewed as they were too unwell to participate. Six were unaware of their diagnosis at interview.

Half (13/26) of the interviewees were ultimately diagnosed with cancer, 9 with pancreatic cancer (3 early stage, 3 late stage, 3 unstaged), and 4 with other cancers (duodenal, gallbladder, oesophageal, lymphoma). The 13 participants with non-cancer diagnoses included hiatus hernia, gallstones and pancreatitis. All participants were interviewed soon after their referral to specialist care (range: 1 day to 18 weeks), with a quarter (6, 23%) prior to diagnosis, half (12, 46%) within 4 weeks of diagnosis, and the remainder within 18 weeks (8, 31%).

### Duration of symptoms

Most participants were able to recall when they first noticed their symptom/s and the date they went to their GP, though for some it was difficult to recall the exact date they decided to seek help. The TTP ranged from 1-270 days, with a median of 21 days. Three individuals were frequent attenders to their GP and so we were unable to define the precise first date of presentation. Two thirds (68%) consulted their GP within 6 weeks of their trigger symptom. While more than half (58%) consulted their GPs only once before referral to secondary care, four patients (16%) saw their GP on more than three occasions.



For peer review only - http://bmjopen.bmj.com/site/about/guidelines.xhtml

**Table 1: Participant characteristics**

| | Cancer diagnosis n=13 (%) | Other conditions diagnosis n= 13 (%) |
|---|---|---|
| **English region** | | |
| Eastern | 10 (77) | 9 (69) |
| North Eastern | 3 (23) | 4 (31) |
| **Gender** | | |
| Female | 6 (46) | 8 (62) |
| Male | 7(54) | 5 (38) |
| **Age** | | |
| Mean Years (range) | 67 (50-84) | 63 (49-84) |
| 40-59 | 4 (31) | 3 (23) |
| 60-69 | 4 (31) | 4 (31) |
| 70-79 | 2 (15) | 4 (31) |
| 80+ | 3(23) | 2 (15) |
| **Ethnicity** | | |
| White British | 13 (100) | 13 (100) |
| **Educational qualifications** | | |
| Higher Education | 3 (23) | 1 (8) |
| Further Education/Secondary school | 6 (46) | 8 (62) |
| None | 4 (31) | 4 (31) |
| **IMD quintiles** | | |
| Least deprived 1 | 4 (31) | 2 (15) |
| 2 | 4 (31) | 4 (31) |
| 3 | 2 (15) | 3 (23) |
| 4 | 3 (23) | 2 (15) |
| Most deprived 5 | 0 (0) | 2 (15) |
| **Diagnoses** | | |
| **Cancer** | | |
| Pancreas | 9 (69) | - |
| Duodenal | 1 (8) | - |
| Gallbladder | 1 (8) | - |
| Oesophageal | 1 (8) | - |
| Lymphoma | 1 (8) | - |
| **Other conditions** | | |
| Hiatus Hernia | - | 3 (23) |
| Gallstones | - | 2 (15) |
| Pancreatitis | - | 2 (15) |
| Others | - | 6 (46) |
| **GP appointments made** | | |
| 0* | 0 (0) | 2 (15) |
| 1 | 9 (62) | 6 (46) |
| 2 | 1 (8) | 4 (31) |
| *emergency presentation 3+ | 3 (23) | 1 (8) |

For peer review only

**Qualitative themes**

We were unable to find any clear differences regarding symptom appraisal or help-seeking between the accounts of people diagnosed with cancer and those with other conditions. We therefore report the findings together. We were also unable to find any clear differences according to whether or not participants knew their diagnosis at the time of the interview, or their characteristics including gender, age and region. Extracts from interviews illustrate the findings: each quotation is contextualised, by the patient's gender (M/F), age (40-59, 60-69, 70-79, 80+), TTP (weeks), and diagnosis (cancer, non-cancer).

For peer review only

**<u>Theme 1: Initial appraisal of symptoms</u>**

Two main sub-themes emerged as participants described how they first noticed and initially interpreted their symptoms, and often attributed them to a common non-serious cause or condition.

***1.1 Bodily changes***

People mainly described initial subtle or non-specific bodily changes such as feeling tired or 'different'. These changes were often intermittent and insidious in their development.

*"I've had this sort of tiredness and a little bit of discomfort on and off for a time"* (M, 70-79, 13-26 weeks, cancer)

*"I've felt a bit like as if I've got a nervous tummy, like it gurgles up…"* (F 50-59, regular attender to GP, non-cancer)

People also described subtle changes in appetite: *"Well I went off my food a bit I think, my daughter said I went off my food a bit"* (F, 80+yrs, 2-4 weeks, cancer)

***1.2 Symptom attributions (Table 2)***

Initial explanations for these bodily changes or symptoms mainly related to diet and other daily activities. Nine participants with other conditions, particularly gastro-intestinal conditions such as gastro-oesophageal reflux disease, often appraised their new symptoms against their "usual" symptoms, and whether their treatment was still effective. This group tended to downplay their symptoms. Some people also attributed these bodily changes or symptoms to other medical conditions or side-effects of their medication. Others 'normalised' their initial symptoms to being caused by aging, common conditions such as a virus or common cold, indigestion or a urine infection.

These explanations were described in the rich context of people's lives, such as being busy, caring for others, or the time of the year. When considered in these contexts, most people initially felt that their symptoms were acceptable and did not warrant seeking help:



For peer review only - http://bmjopen.bmj.com/site/about/guidelines.xhtml

*"I thought I was a bit tired-er than normal, but I mean, I was tearing around. My daughter was coming back for three months and they've got a house in the next village, I was over there doing things in the house, getting it ready."* (F, 60-69, 2-4 weeks, cancer)

**Table 2: Illustrative quotations relating to Symptom Attribution, a sub-theme of Theme 1- Initial Appraisal of Symptoms**

| | |
|---|---|
| Diet and lifestyle | *"It comes and goes, it must be something that I eat and it just doesn't agree with me"* (F, 60-69, 13-26 weeks, non-cancer)<br><br>*"I think it's the picking up, it's the bending that does it a lot with my job as well, 'cos I lift heavy (clothing) all day, and I'm putting them over people and then bending and sitting down"* (F, 40-59, regular attender to GP, cancer) |
| Existing gastrointestinal conditions | *"I've always suffered with what's it called, you know, reflux, over the years, sometimes I could take some indigestion remedy and that Gaviscon thing and it'd calm down"* (F, 40-49, <2 weeks, non-cancer) |
| Other medical conditions, side-effects of medication | *"At first I started putting it down to the diabetic pills, I thought 'Well it's probably because I've changed those', you know, and this Pioglitazone, I kept thinking 'Well it's that',* (F 60-69, 2-4 weeks, cancer)<br><br>*"I've had this sort of tiredness and a little bit of discomfort on and off for a time, I thought it was possibly diabetes…it kept on… and we thought was perhaps as a result of some of the medication I was taking for other things."* (M, 70-79, 13-26 weeks, cancer) |

<ins>**Theme 2: Responses to initial symptom appraisal**</ins>

Participants reported a number of responses to initial symptom appraisal.

***2.1 Lifestyle adaptations***

In the light of these attributions and explanations, some people began to make adaptations to their diet or daily living activities to alleviate their symptoms; in some cases, these changes were quite significant:

*"If I had smaller meals, but slightly more frequently, I could actually control keeping food, you know, keeping comfortable"* (M, 60-69, 5-8 weeks, cancer)

*"I think you learn to live with it and you just adapt to it and start to make the changes so it doesn't happen…. I think what had happened with me is I couldn't control it any longer, no matter if I stopped eating things, didn't eat out, it was happening anyway"* (F, 60-69, 26-52 weeks, non-cancer)

***2.2 Role of others in appraisal and adaptations***

Some people discussed their early symptoms with close relatives, particularly partners or children. This could prompt discussions around potential explanations



For peer review only - http://bmjopen.bmj.com/site/about/guidelines.xhtml

and suggestions for improving symptoms, such as changes in content or timing of meals; at times it could also lead to delays in helpseeking:

*"When I mentioned to my wife the colour of my stools, which was the first symptom, she said I wasn't getting enough carbohydrates or protein or something…something wasn't right in my diet. So I changed my diet, I was eating fruit and vegetables and things like that, a lot more, and it still stayed the same."* (M, 50-59, 13-26 weeks, cancer)

**Theme 3: Further appraisal of symptoms**

Changes in patterns of symptoms often led participants to further appraise their symptoms, and to question the reasons for their occurrence and their original explanations.

***3.1 Symptom progression (Table 3)***

Symptoms became of greater concern when they became more severe or frequent, or when further subtle symptoms developed. In some cases, it appeared that there was progression from subtle and intermittent symptoms of a manageable and tolerable nature, to symptoms which were more alarming such as pain. Across the whole sample, people described pain in a number of different parts of the body including their shoulder, chest, upper abdomen and back.

Another man described the chronology of his symptoms; having started with mild indigestion symptoms, additional, more alarming, symptoms developed: *"gradually… the stools over a period of time, but the urine it seemed to happen more or less in a week, from normal colour to really deep colour"* (M, 50-59, 13-26 weeks, cancer)

Many participants reported subtle yet progressive changes to their appetite and enjoyment of food. Weight loss as a result of changes in appetite was also reported by a few participants.

***3.2 Symptom monitoring and self-management***

The subtlety and slow progression of symptoms prompted some participants to monitor their symptoms, and some to consider alternative self-management strategies. Many used over-the-counter remedies to attempt to alleviate their symptoms of indigestion or abdominal pain:

*"It was only when I started feeling a bit of strangeness that I decided to try and keep a pattern of them"* (F, 60-69, 2-4 weeks, cancer)

***3.3 Impact on life***

Most people described how their symptoms gradually or rapidly impacted on their quality of life and daily routine, including their social activities and hobbies, as well as their ability to sleep. Some felt that they were less able to complete tasks at home; for others, work became more challenging:

*"I decided I'd got this burning pain and I noticed that I was beginning to get a little bit short of breath, and also I couldn't do the same things I used to, like I found digging was becoming impossible"* (M, 70-79, 13-26 weeks, non-cancer)

***3.4 Previous family and personal history of cancer***



For peer review only - http://bmjopen.bmj.com/site/about/guidelines.xhtml

A few people expressed a concern that they might develop cancer because they had lost a close relative to the disease; one had also previously suffered from cancer. These people were concerned about their personal risk of cancer and reported that they often considered whether their symptoms matched those which they expected from having cancer. Conversely one woman, subsequently diagnosed with a benign condition, had not considered cancer as a possibility because her symptoms did not match her expectations of cancer:

*"I knew I'm generally healthy, I mean I've been going swimming and to the gym…it wasn't seriously debilitating…I expect when you have cancer that you have more symptoms"* (F, 60-69, 26-52 weeks, non-cancer)

**Table 3: Illustrative quotations relating to Symptom Progression, a sub-theme of Theme 3- Further Appraisal of Symptoms**

| | |
|---|---|
| Pain | *"Well, it varied, you know, sometimes I'd have the pain all day and that's very wearing and painful…It's just under the rib cage and it's slightly to the right hand side, just slightly there, always in the same place."* (F, 70-79, <2 weeks, non-cancer)<br><br>*"…I just had a nagging, more like a dull ache, underneath my bust and I thought it was indigestion and it didn't seem to go away, it didn't make any difference whether I ate or not, it was there quite a lot of the time"* (F, 70-79, 2-4 weeks, cancer)<br><br>*"After Christmas that then became all day, every day. I was going to bed with a stomach ache and I was waking up with a stomach ache…It was there all the while and you know, it didn't make sense that I'd have a problem all the while so I thought I was imagining it"* (M, 60-69, 2-4 weeks, cancer) |
| Progressive changes in appetite and enjoyment of food | *"I was actually going off the actual taste of food, I wasn't enjoying food anymore, things that I would normally eat I wasn't enjoying"* (M, 60-69, 5-8 weeks, cancer)<br><br>*"Well it became quite traumatic at one point, when I just [did] not want an evening meal at all. I always eat a reasonable breakfast, but for the evening meal I had no appetite"* (M, 80+, <2 weeks, cancer)<br><br>*… and I was eating less and smaller meals and being really careful what I ate, and I thought "it's good for you but you shouldn't have to be that precise when you eat"* (F, 60-69, 26-52 weeks, non-cancer) |
| Weight loss | *"I was also suffering uncomfortable, you know, let's say stomach irritation, indigestion, wasn't happy eating, so I literally went along to say 'look, this isn't right, I don't mind losing weight but not like this"* (M 60-69, 5-8 weeks, cancer)<br><br>*"I'd probably lost about four pounds which was concerning me but then I thought, you've reduced the amount that you're eating…"* (F 50-59, 2-4 weeks, non-cancer) |

For peer review only

**Theme 4: Deciding to seek help (Table 4)**

The further appraisal of symptoms eventually led most participants to decide to seek help from primary care, although several had emergency visits to A&E.

***4.1 Triggers***

Triggers to seek help usually involved an increased frequency or intensity of symptoms, although sometimes it was precipitated by an alarming event, usually pain. Pain was often the "tipping point" to seek help against a background of other more subtle symptoms. For those without pain, triggers were often complex, and could involve interpreting new or changing symptoms alongside symptoms from other existing conditions and, in some instances, symptoms from other acute illnesses. Healthcare factors, such as availability of a GP appointment, were seldom mentioned.

***4.2 Legitimising help-seeking***

People were often encouraged to seek help by their spouse, family members and friends; they were particularly influenced by comments about changes in their general wellbeing, or recall of specific symptom episodes. A few participants reported considering whether their symptoms had become a 'legitimate' reason to seek healthcare when self-management strategies became ineffective; they tended to refer to the length of time symptoms had been experienced, and their reduced ability to cope.

**Table 4: Illustrative quotations relating to Theme 4: Deciding to Seek Help**

| | Illustrative quotations |
|---|---|
| Pain on a background of more subtle symptoms | *"Sometimes I could take some indigestion remedy and.. it'd calm down, but this were a different kind of pain, this were, oh it's excruciating pain…but that were the first time that… I thought there's something not right here and I need to get some help."* (F, 40-49, <2 weeks, non-cancer) |
| Help-seeking encouraged by others | *"The kids had been saying "mum you're always ill" and I thought "oh yeah, I am". Because you, I think you sort of drift, you get used to things and you don't see any problem with it, and when other people start to say, you know "hang on" and then I thought "oh yeah"*(F, 60-69, 26-52 weeks, non-cancer)<br><br>*"I'd fall asleep on my desk, you know, this isn't me, and other people said "oh you're losing weight"… people actually were saying "you don't look well". It's another lever to go and see, to get it sorted"* (M, 60-69, 5-8 weeks, cancer) |
| Legitimate reason | *"I think my basic problem was pain… from arthritis that I'd had for some time, but it was proving impossible to control it by the methods I was using"* (M 80-89, 2-4 weeks, cancer) |

**DISCUSSION**



For peer review only - http://bmjopen.bmj.com/site/about/guidelines.xhtml

This is the first study to investigate symptom appraisal and help-seeking in patients newly referred to hospital with symptoms suggestive of pancreatic cancer. Due to their subtle and intermittent pattern, many people initially just monitored their symptoms. Symptoms were interpreted based on previous experiences with common conditions such as gastroenteritis, or impact of dietary change or existing gastro-intestinal conditions. Many made changes to their eating patterns including type of food, and frequency and quantity of meals, or managed their symptoms with over-the-counter medication. Consequently, they tended not to go to their GP at that point. Changes to symptom frequency, duration or severity, their impact on daily life, or the appearance of additional symptoms, particularly pain, led many people to further appraise their symptoms and visit their GP. Family and friends often made important contributions to encouraging help-seeking by reflecting on changes in symptoms, although at times social contacts could re-inforce self-management strategies. These patterns of symptom appraisal and help-seeking were seen regardless of final diagnosis.

**Strengths and limitations**

The major strength of this study is that we prospectively recruited patients with symptoms suggestive of pancreatic cancer, and interviewed them during their pathway to diagnosis and treatment. This allowed detailed discussions about factors affecting symptom appraisal and help-seeking while they were still fresh events, and greatly reduced the possibility of *post-hoc* rationalisation and recall bias. We have used similar approaches in interview studies with people recently diagnosed with melanoma [22], and people with symptoms suggestive of lung [10] and colorectal [12] cancers. Recruiting patients during their diagnostic pathway enabled comparisons about symptom appraisal and help-seeking between people subsequently diagnosed with cancer and people with non-cancer conditions; importantly, we were not able to find differences between them. Our recruitment process meant that some patients were subsequently diagnosed with a cancer other than pancreatic cancer. We do not consider this a weakness of the study; rather, it serves to highlight the complexity and challenges of diagnosing pancreatic cancer, and how similarly a range of upper gastro-intestinal cancers can present. While we aimed to recruit equal numbers of patients with pancreatic cancer and benign conditions, several patients with cancer withdrew before interview as they were too unwell.

Our approach to data collection and analysis was strengthened by the use of a strong theoretical framework: underpinned by recommendations from the Aarhus statement on improving design and reporting studies on early cancer diagnosis [18], we applied the framework of the widely used Model of Pathways to Treatment [16,17], and a specifically-developed calendar-landmarking instrument which aided



For peer review only - http://bmjopen.bmj.com/site/about/guidelines.xhtml

some participants' recall of dates of their symptoms and help-seeking [14]. Other strengths include recruitment from two English regions and a number of hospitals to ensure patients from a wide range of socio-economic backgrounds, and who had entered hospital care via a variety of routes, enabling us to gather a broad range of experiences. Finally, the research team had a broad range of scientific and clinical expertise, and we sought input from our lay members at all stages of the research process, including analysis and interpretation of the data.

While we acknowledge that these experiences may not be representative of all people with symptoms suggestive of pancreatic cancer, the demographics of this cohort are similar to that of the main SYMPTOM-Pancreas study [12]. Although a major finding was that there were similar patterns in symptom appraisal and help-seeking between those diagnosed with cancer and those with other diagnoses, this may have been partly due to recruiting patients relatively late in their diagnostic pathway, when GPs had referred with a suspicion of pancreatic cancer. Ideally we would have recruited people with similar symptoms from earlier in their diagnostic pathway and from primary care, but there are significant challenges with the feasibility and costs of recruiting such a cohort containing sufficient cases of pancreatic cancer. The presence of relatives in some interviews may have led to potential bias in the participants' accounts and recollections, but this was minimised by only including participant's words in the analysis. Finally, we can only report the experiences shared with the researchers at the time of the interview: there may have been events which were not discussed if they were felt to be too emotional or private.

**Comparison with existing literature**

Although these findings are in line with the broader literature investigating help-seeking for cancer symptoms [10,12, 22], very little has been published on symptom appraisal and help-seeking among patients diagnosed with pancreatic cancer. Evans *et al.* recently reported the only other similar study, also set in England, and highlighted the intermittent and subtle nature of symptoms with their title: '"It can't be very important because it comes and goes"- patients' accounts of intermittent symptoms preceding a pancreatic cancer diagnosis' [8]. However, their interviews were conducted with people up to several years after a diagnosis of pancreatic cancer, and included data from interviews with relatives of people who had died. Our findings are strengthened by interviewing patients earlier in their illness pathway, thereby capturing subtle assessments of initial bodily changes and explanations, and minimising post-hoc rationalisation and recall bias. We could not identify any subtle differences in the nature of symptoms or help-seeking between those who eventually were found to have pancreatic cancer, and those who were not. This is consistent with the quantitative findings of the SYMPTOM-pancreas study



For peer review only - http://bmjopen.bmj.com/site/about/guidelines.xhtml

[9], where the evolution of additional symptoms such as fatigue, change in bowel habit, weight loss, decreased appetite and jaundice were associated with pancreatic cancer.

Other studies of pancreatic cancer symptoms have mainly used quantitative retrospective designs. For example, Stapley *et al*. undertook a case-control study using routinely recorded UK primary care data which demonstrated how poorly predictive most symptoms are of pancreatic cancer in primary care populations. While the positive predictive value of jaundice was relatively high (21%), it was much lower for other symptoms including abdominal and back pain, nausea, vomiting, change in bowel habit, weight loss and malaise (<1%) [6]. Keane *et al*. more recently used another large UK primary care database to investigate the early symptom profiles of patients diagnosed with pancreatic ductal or biliary adenocarcinoma and found a similar wide range of early symptoms [23]. They also found that, in the year prior to diagnosis, these patients visited their GP on a median of 18 (IQR 11–27) occasions, which resonated with findings from another large English database study showing that 41% of patients diagnosed with pancreatic cancer had visited their GP at least three times before referral to specialist care [7]. In contrast, more than half our sample reported only visiting their GP once before referral, suggesting either that our sample were recruited early in their symptom pathway, or perhaps that they had symptoms such as jaundice which are more likely to prompt early referral. Other quantitative studies set among patients newly diagnosed with pancreatic cancer patients have reported fatigue, dyspepsia and subtle changes in appetite such as early satiety in the preceding six months, confirming our finding that there may be a window of opportunity for timely investigations when patients report combinations of non-specific or subtle symptoms [4,24,25].

**Implications for research and practice**

Our findings add to current knowledge about the subtle and non-specific nature of early symptoms of pancreatic cancer, and enhance our understanding of their often intermittent and insidious nature. They highlight the importance of on-going, and often iterative, appraisal of symptoms as described in the Model of Pathways to Treatment [16,17]. The findings have characterised pain as a 'tipping point' for presentation to healthcare, and underpinned the importance of family and friends in symptom monitoring, recognising evolving and new symptoms and endorsement of help-seeking [26]. The challenge for symptom awareness campaigns is to promote earlier presentation with these more subtle and intermittent symptoms which lead to changes in eating patterns including type of food, and frequency and quantity of meals, and weight loss. The 'Know 4 Sure' campaign was one of the first to raise awareness of non-site-specific symptoms including weight loss, pain, lump and unexplained bleeding. It was associated with increase in referrals for a range of



For peer review only - http://bmjopen.bmj.com/site/about/guidelines.xhtml

cancers including upper GI cancer [27]. Whether public health campaigns can effectively promote messages about combinations of non-specific symptoms is unknown, but may be important in pancreatic cancer.

The newly revised NICE guidance on Suspected Cancer: Recognition and Referral [28] supports a lower threshold for investigating and referring people with single and multiple symptoms suggestive of pancreatic cancer, particularly those in higher risk groups such as newly diagnosed diabetics. The findings from this study could inform GP education approaches to promote timely investigation and referral for people with these non-specific and intermittent symptoms. These numerous subtle and non-specific symptoms, when combined with abnormal blood tests and the use of clinical decision support tools, might alert GPs to a possible pancreatic cancer diagnosis earlier. Safety-netting, defined as is a diagnostic strategy or consultation technique to help manage diagnostic uncertainty and ensure that patients are followed up in a timely and appropriate manner, is also important due to the evolving nature of symptoms; appropriate strategies would include symptom monitoring and early review.

In conclusion, this study has described and contextualised the appraisal and help-seeking behaviours of patients with symptoms that could represent pancreatic cancer. Greater awareness of combinations of the subtle and intermittent symptoms, and their evolving nature, is needed to prompt timelier help-seeking and investigation among people with symptoms of pancreatic cancer.

For peer review only



For peer review only - http://bmjopen.bmj.com/site/about/guidelines.xhtml

**Acknowledgments**

We thank the patients who so kindly and freely gave their time and experiences to this study. We thank Helen Morris the SYMPTOM study manager, our other study patient and public representatives Sue Ballard and Victor Boulter, and the staff at the participating hospitals (Bedford Hospital NHS Trust, Cambridge University Hospitals NHS Foundation Trust; Hinchingbooke Healthcare NHS Trust, The Queen Elizabeth Hospital Kings Lynn NHS Trust, Peterborough and Stamford NHS Foundation Trust, University Hospital of North Durham; Darlington Memorial Hospital: University Hospital of North Tees, West Suffolk NHS Foundation Trust) who facilitated participant recruitment and supported the study. The authors would also like to acknowledge the contribution made by the NIHR funded Discovery Programme Steering Committee comprising: Roger Jones (chair); Alison Clutterbuck; Ardiana Gjini; Joanne Hartland; Maire Justice; Jenny Knowles; Richard Neal; Peter Rose.

**Contributors**
FMW, JE, GR and WH were responsible for study design, study conduct and management. KM, LB and NH collected the data, and with the support of JB, JE, JL, MJ and FMW, KM analysed and interpreted the data. KM led on the manuscript preparation. All authors contributed to the writing and completion of the paper and have approved the final the manuscript.

**Funding**
This paper presents independent research funded by the National Institute for Health Research Programme Grants for Applied Research programme (RP-PG-0608-10045) and Pancreatic Cancer Action (registered charity 1137689). The views expressed are those of the authors and not necessarily those of the NHS, the NIHR or the Department of Health. The funders of this study had no role in study design, data collection, analysis, and interpretation, or the writing of this research paper. FMW is supported by an NIHR Clinician Scientist award.

**Competing interests**
The authors have no conflicts of interest to declare.

**Data sharing statement**
No additional data are available.

**Ethics approval**
Ethical approval was granted by the Cambridgeshire 3 Research Ethics Committee (10/H0306/50).



For peer review only - http://bmjopen.bmj.com/site/about/guidelines.xhtml

**REFERENCES**


1. Cancer Research UK, *Incidence Statistics* 2011. Retrieved from http://www.cancerresearchuk.org/health-professional/cancer-statistics/incidence/common-cancers-compared#heading-Zero (Last accessed 31.05.16).

2. Hiom S. Diagnosing cancer earlier: reviewing the evidence for improving cancer survival. *Br J Cancer* 2015; 112 (supp1): S1-S5.

3. Neal RD, Tharmanathan P, France B et al. Is increased time to diagnosis and treatment in symptomatic cancer associated with poorer outcomes? Systematic review. Br J Cancer 2015; 112:S92-S107.

4. Holly EA, Chaliha I, Bracci PM, et al. Signs and symptoms of pancreatic cancer: a population-based case-control study in the San Francisco Bay area. *Clin Gastroenterol Hepatol* 2004; 2:510-7.

5. Hippisley-Cox J, Coupland C. Identifying patients with suspected pancreatic cancer in primary care: derivation and validation of an algorithm. *Br J Gen Pract* 2012; 62: e38-45.

6. Stapley S, Peters TJ, Neal RD, et al. The risk of pancreatic cancer in symptomatic patients in primary care: a large case-control study using electronic records. *Br J Cancer* 2012; 106: 1940-4.

7. Lyratzopoulos G, Neal RD, Barbiere JM, et al. Variation in number of general practitioner consultations before hospital referral for cancer: findings from the 2010 National Cancer Patient Experience Survey in England, *The Lancet Oncology* 2012; 13: 353–365.

8. Evans J, Chapple A, Salisbury H, et al. "It can't be very important because it comes and goes"--patients' accounts of intermittent symptoms preceding a pancreatic cancer diagnosis: a qualitative study. *BMJ Open* 2014; 4: e004215.

9. Birt L, Hall N, Emery J, et al. Responding to symptoms suggestive of lung cancer: a qualitative interview study. *BMJ Open Respir Res* 2014; 1(1): e000067.

10. Walter FM, Rubin G, Bankhead C, et al. Symptoms and other factors associated with time to diagnosis and stage of lung cancer: a prospective cohort study. *Br J Cancer* 2015; 112 (supp1): S6-S13.

11. Hall N, Birt L, Banks J et al. Symptom appraisal and healthcare-seeking for symptoms suggestive of colorectal cancer: a qualitative study. *BMJ Open* 2015;5(10):e008448.

12. Walter FM, Mills K, Mendonca SC, et al. Symptoms and patient factors associated with diagnostic intervals for pancreatic cancer (SYMPTOM pancreatic study): a prospective cohort study. *Lancet Gastroenterol Hepatol* 2016; 1:298-306.




For peer review only - http://bmjopen.bmj.com/site/about/guidelines.xhtml

13. Emery JD, Walter FM, Gray V, et al. Diagnosing cancer in the bush: a mixed methods study of GP and specialist diagnostic intervals in rural Western Australia. *Fam Pract* 2013; 30(5):541-50.

14. Mills K, Emery J, Cheung C, et al. A qualitative exploration of the use of calendar landmarking instruments in cancer symptom research. BMC *Fam Pract* 2014; 15:167.

15. Guest G, Bunce A, Johnson L. How many interviews are enough? An experiment in data saturation and variability. *Field Methods* 2006; 18:59-82.

16. Walter FM, Scott SE, Webster A, et al. The Andersen Model of Total Patient Delay: a systematic review of its application in cancer diagnosis. *J Health Services Research & Policy* 2012; 17(2):110-8.

17. Scott SE, Walter FM, Webster A et al. The model of pathways to treatment: conceptualization and integration with existing theory. *Br J Health Psychol* 2013; 18(1): 45-65.

18. Weller D, Vedsted P, Rubin G, et al. The Aarhus statement: improving design and reporting of studies on early cancer diagnosis. *Br J Cancer* 2012; 106(7):1262-1267.

19. Braun, V. and Clarke, V. Using thematic analysis in psychology. Qualitative Research in Psychology, 2006, 3 (2). pp. 77-101.

20. Gale NK, Heath G, Cameron E, Rashid S, Redwood S. Using the framework method for the analysis of qualitative data in multi-disciplinary health research, BMC Med Res Methodol. 2013 Sep 18;13:117.

21. Ritchie J, Spencer L. Qualitative data analysis for applied policy research. In: Bryman A, Burgess R, eds. *Analysing qualitative data*. London: Routledge, 1993:173-94.

22. Walter FM, Birt L, Cavers D, et al. This isn't what mine looked like: a qualitative study of symptom appraisal and help-seeking in people recently diagnosed with melanoma. *BMJ Open* 2014; 21: 4(7) e005566.

23. Keane MG, Horsfall L, Rait G, Pereira SP. A case-control study comparing the incidence of early symptoms in pancreatic and biliary tract cancer. *BMJ Open* 4:e005720.

24. Gullo L, Tomassetti P, Migliori M, et al. Do early symptoms of pancreatic cancer exist that can allow an earlier diagnosis? *Pancreas* 2001; 22:210-3.

25. Ingeman ML, Christensen MB, Bro F. The Danish cancer pathway for patients with serious non-specific symptoms and signs of cancer–a cross-sectional study of patient characteristics and cancer probability, *BMC Cancer* 2015; 15:421.



For peer review only - http://bmjopen.bmj.com/site/about/guidelines.xhtml

26. Leydon GM, Bynoe-Sutherland J, Coleman MP. The journey towards a cancer diagnosis: the experiences of people with cancer, their family and carers. *Eur J Cancer Care* 2003; 12:317-326.

27. Cancer Research UK, "Know 4 sure" campaign 2014. Retrieved from http://www.cancerresearchuk.org/health-professional/early-diagnosis-activities/be-clear-on-cancer/know-4-sure-campaign (Last accessed 31.05.2016).

28. National Institute for Health and Care Excellence, Suspected Cancer: Recognition and Referral 2015. Retrieved from http://www.nice.org.uk/guidance/ng12 (Last accessed 31.05.16).

For peer review only



For peer review only - http://bmjopen.bmj.com/site/about/guidelines.xhtml

# The SYMPTOM Study

## Interview Topic Guide

1. Welcome and introduction

2. Confirm understanding of study, participation & recording, confidentiality issues & consent

3. Can you tell me about what first prompted you to go to your doctor?

4. Can you describe the symptom(s) a bit more?
   *[Prompts: location, duration, severity, ever had this before?]*

5. You mentioned (symptom(s)), were there any more symptoms that you noticed about this time?
   *[Prompt: try to identify all participant-reported symptoms first; the checklist comes later]*

6. What did you do to cope with/help these symptoms?
   *[Prompts: How long did it take before you decided to use any treatment? How long did you try it for? Did it help at all?]*

7. So, please could you help me try and record the number and chronology/timing of each symptom?
   a. *Using the Calendar landmarking tool (see below)*
   b. *By drawing a timeline of each symptom with the participant*
   c. *By using the model (attached) to identify appraisal, help-seeking, and diagnostic intervals.*

7. Now, we've talked about the symptoms that you have described: I'd also like to check whether you have had any of the following symptoms?
   *[Prompt for checklist, and try to draw the timeline for each symptom: Lung symptoms: e.g. coughing up blood, dry/productive cough, chest tightness/pain, hoarse voice etc.; Colorectal & pancreas symptoms: e.g. abdominal pain, change of bowel habit, rectal bleeding, yellow jaundice etc.; General symptoms: e.g. increased fatigue, loss of appetite, weight loss etc.]*

9. You went to see your doctor about your symptoms: did you have any thoughts about what might have been wrong?

10. Did you discuss your symptoms with anyone other than your Doctor?
    *[Prompts: Who were they? Did they encourage/discourage you to seek medical advice?]*

11. Do you know of anyone who has also had any similar symptoms or investigations to you?
    *[Prompts: relationship, symptoms they had, care they received, outcome etc]*

12. Can you tell me about the first appointment with your doctor for these symptoms we've been talking about?
    *[Prompts: Was there a specific event that made you make the appointment? What was the trigger? Had you waited for any reason?]*

13. What was discussed at that first appointment?
    *[Prompts: Did you have any thoughts about what would happen when you first went to see your GP? What happened– were you examined? Did the GP arrange any tests or referrals?]*

14. How did you feel about the outcome of the consultation?
    *[Prompt: did you feel you were taken seriously by the GP?]*

15. Did you see the GP for this symptom more than once before you were referred to the


For peer review only - http://bmjopen.bmj.com/site/about/guidelines.xhtml

hospital?
*[Prompt: each visit may need unpacking for symptoms discussed, feelings about the consultation outcomes etc.]*

*16.* In your opinion, do you feel there was anything that prolonged you finding out about what is causing your symptoms [*Prompts: information about the symptoms associated with serious conditions, difficulty in getting an appointment, time stresses from work/family etc., medical staff attitudes etc.]*

17. Have you experienced any symptoms or feelings or sensations that you *didn't* talk to the GP about, even if you just felt "a bit off".
*[Prompt: Was there a reason why you did not mention this to your doctor?]*

*18. Now we've discussed your symptoms, I'd like to ask you a few questions about being referred and the test[s] you've had. Thinking back to your first consultation with your doctor, can you tell me what tests or investigations were mentioned (CXR, colonoscopy etc). When you were referred for the test can you tell me-?*

A. What information the GP provided about this test
   - *[Prompts: info provided – face to face, info leaflet, website]*

B. Did you understand why you were being referred/tested and what it involved?
   - *[Prompts: knowledge of physical aspects of test, possible side effects, meaning of results - diagnosis, necessity of further tests]*

C. Do you feel that you made a conscious decision to have this test or do you feel that the decision was made for you by the GP
   - *[Prompts: feel involved in decision to be tested, conscious decision – why?]*
   - *[Prompts: accepted referral – why?; Conscious decision – why?]*
   - *[Prompts: consider not having test?]*
   - *[Prompts: discuss test with family/friends – did this influence?]*

D. How did you feel about being referred for this test
   - *[Prompts: advantages - improved prognosis, wider treatment options, reassurance; disadvantages - physical & emotional challenges, practicalities, any other aspects]*

E. Can you tell us what happened during this test
   - *[Prompts: Did you take a family member/ friend with you? what happened after, how information was communicated, how test differed from expectations]*

F. Can you tell us about the practicalities of taking this test?
   - *[Prompts: time off work – how long, manage family commitments & childcare]*

G. Do you think that all patients with symptoms like yours should have these tests?

19. We've been through a lot today but is there anything else you'd like to discuss?

20. Thanks, confirmation of consent and confidentiality.

For peer review only


For peer review only - http://bmjopen.bmj.com/site/about/guidelines.xhtml

# Consolidated criteria for reporting qualitative studies (COREQ): 32-item checklist

Developed from:
Tong A, Sainsbury P, Craig J. Consolidated criteria for reporting qualitative research (COREQ): a 32-item checklist for interviews and focus groups. *International Journal for Quality in Health Care*. 2007. Volume 19, Number 6: pp. 349 – 357

**YOU MUST PROVIDE A RESPONSE FOR ALL ITEMS. ENTER N/A IF NOT APPLICABLE**

| No. Item | Guide questions/description | Reported on Page # |
|---|---|---|
| **Domain 1: Research team and reflexivity** | | |
| *Personal Characteristics* | | |
| 1. Interviewer/facilitator | Which author/s conducted the interview or focus group? | Undertaken by K Mills, N Hall and L Birt –data collection section reported p6. |
| 2. Credentials | What were the researcher's credentials? E.g. PhD, MD | All have PhD and extensive qualitative research experience, see p6. |
| 3. Occupation | What was their occupation at the time of the study? | All were Research Associates, see p1-2. |
| 4. Gender | Was the researcher male or female? | All female. |
| 5. Experience and training | What experience or training did the researcher have? | See p6. |
| *Relationship with participants* | | |
| 6. Relationship established | Was a relationship established prior to study commencement? | No. |
| 7. Participant knowledge of the interviewer | What did the participants know about the researcher? e.g. personal goals, reasons for doing the research | Participants had received the Patient Information Sheet, see p6. |
| 8. Interviewer characteristics | What characteristics were reported about the interviewer/facilitator? e.g. Bias, assumptions, reasons and interests in the research topic | Reported under Methods, analytic processes, see p7. |
| **Domain 2: study design** | | |
| *Theoretical framework* | | |
| 9. Methodological orientation and Theory | What methodological orientation was stated to underpin the study? e.g. grounded theory, discourse analysis, | We used thematic analysis, see p7. |

For peer review only

| | | |
|---|---|---|
| | ethnography, phenomenology, content analysis | |
| *Participant selection* | | |
| 10. Sampling | How were participants selected? e.g. purposive, convenience, consecutive, snowball | Using purposive sampling, see p8. |
| 11. Method of approach | How were participants approached? e.g. face-to-face, telephone, mail, email | Recent referral to hospital, reported under recruitment section, see p6. |
| 12. Sample size | How many participants were in the study? | 26 interviews, reported on p8. |
| 13. Non-participation | How many people refused to participate or dropped out? Reasons? | 34 individuals were purposefully sampled and invited to participate. 8 were not interviewed as they were not well enough to complete the interview, described on p8. |
| *Setting* | | |
| 14. Setting of data collection | Where was the data collected? e.g. home, clinic, workplace | Home, reported on p6. |
| 15. Presence of non-participants | Was anyone else present besides the participants and researchers? | Relatives were present in a few of the interviews, reported on p6-7. |
| 16. Description of sample | What are the important characteristics of the sample? e.g. demographic data, date | See Table 1. |
| *Data collection* | | |
| 17. Interview guide | Were questions, prompts, guides provided by the authors? Was it pilot tested? | Topic guide used, similar to that used for the other SYMPTOM studies with participants with symptoms suggestive of lung and colorectal cancer (appendix) |
| 18. Repeat interviews | Were repeat inter views carried out? If yes, how many? | No |
| 19. Audio/visual recording | Did the research use audio or visual recording to collect the data? | Yes, audio-recording, see p7. |
| 20. Field notes | Were field notes made during and/or after the inter view or focus group? | Personal notes were kept, but |

For peer review only

| | | |
|---|---|---|
| | | they were not included in the analysis. |
| 21. Duration | What was the duration of the inter views or focus group? | 40-70 minutes, see p6. |
| 22. Data saturation | Was data saturation discussed? | Yes, see p7. |
| 23. Transcripts returned | Were transcripts returned to participants for comment and/or correction? | No, the authors did not consider respondent validation to be an appropriate approach in this patient group. |
| **Domain 3: analysis and findings** | | |
| *Data analysis* | | |
| 24. Number of data coders | How many data coders coded the data? | Three- KM, NH & LM coded the data, see p6. |
| 25. Description of the coding tree | Did authors provide a description of the coding tree? | No |

| | | |
|---|---|---|
| 26. Derivation of themes | Were themes identified in advance or derived from the data? | Initially inductively using data-driven coding, then deductively, using the framework of the Model of Pathways to Treatment, see p7. |
| 27. Software | What software, if applicable, was used to manage the data? | NVivo- reported on p8. |
| 28. Participant checking | Did participants provide feedback on the findings? | No, although some validation was provided by Steering group including 2 lay members who have been included as authors, reported on p7. |
| *Reporting* | | |
| 29. Quotations presented | Were participant quotations presented to illustrate the themes/findings? Was each quotation identified? e.g. participant number | Yes throughout results in the text and in Tables 2,3, and 4, reported pp 10-14. |

For peer review only

| | | |
|---|---|---|
| 30. Data and findings consistent | Was there consistency between the data presented and the findings? | Yes. |
| 31. Clarity of major themes | Were major themes clearly presented in the findings? | Yes, see pp10-14. |
| 32. Clarity of minor themes | Is there a description of diverse cases or discussion of minor themes? | Yes, see pp 10-14. |

**Once you have completed this checklist, please save a copy and upload it as part of your submission. When requested to do so as part of the upload process, please select the file type: *Checklist*. You will NOT be able to proceed with submission unless the checklist has been uploaded. Please DO NOT include this checklist as part of the main manuscript document. It must be uploaded as a separate file.**

For peer review only

BMJ Open

# Understanding symptom appraisal and help-seeking in people with symptoms suggestive of pancreatic cancer: a qualitative study.



| | |
|---|---|
| Journal: | *BMJ Open* |
| Manuscript ID | bmjopen-2016-015682.R3 |
| Article Type: | Research |
| Date Submitted by the Author: | 27-Jul-2017 |



| | |
|---|---|
| Complete List of Authors: | Mills, Katie; University of Cambridge, Public Health & Primary Care<br>Birt, Linda; University of East Anglia, School of Health Sciences<br>Emery, Jon; University of Melbourne, General Practice and Primary Care Academic Centre<br>Hall, Nicola; Durham University School of Medicine Pharmacy and Health<br>Banks, Jonathan; University of Bristol, Social & Community Medicine<br>Johnson, Margaret ; University of Cambridge, Patient Representative c/o Public Health & Primary Care<br>Lancaster, John; University of Cambridge, Patient Representative c/o Public Health & Primary Care<br>Hamilton, Willie; University of Exeter Medical School, Primary Care Diagnostics<br>Rubin, Greg; Durham University, School of Medicine and Health<br>Walter, Fiona; University of Cambridge, Dept of Public Health and Primary Care |



| | |
|---|---|
| <b>Primary Subject Heading</b>: | Qualitative research |
| Secondary Subject Heading: | General practice / Family practice, Gastroenterology and hepatology |
| Keywords: | pancreatic cancer, diagnosis, appraisal, help-seeking, symptoms |
| | |

SCHOLARONE™ Manuscripts

# Understanding symptom appraisal and help-seeking in people with symptoms suggestive of pancreatic cancer: a qualitative study.

**Author names, affiliations, full address and postcode, contact details:**

**Katie Mills (Corresponding author**- ko298@medschl.cam.ac.uk)
Research Associate
The Primary Care Unit
Department of Public Health and Primary Care
University of Cambridge
Cambridge CB1 8RN

**Linda Birt** Linda.Birt@uea.ac.uk
At the time of completing this research:
Research Associate
The Primary Care Unit
Department of Public Health and Primary Care
University of Cambridge
Cambridge CB1 8RN

**Jon D Emery** jon.emery@unimelb.edu.au
Herman Professor of Primary Care Cancer Research
Department of General Practice
University of Melbourne
Australia

**Nicola Hall** n.hall@sunderland.ac.uk
At the time of completing this research:
Research Associate
School of Medicine, Pharmacy & Health
Durham University
Stockton on Tees TS17 6BH

**Jonathan Banks** Jon.Banks@bristol.ac.uk
Research Fellow
School of Social and Community Medicine
University of Bristol
Bristol BS8 2PS

**Margaret Johnson**
Patient representative c/o: ottersriverbank@natsirt.co.uk
Primary Care Unit
Department of Public Health & Primary Care



For peer review only - http://bmjopen.bmj.com/site/about/guidelines.xhtml

University of Cambridge
Cambridge CB1 8RN

**John Lancaster** lancaster366@btinternet.com
Patient representative c/o:
Primary Care Unit
Department of Public Health & Primary Care
University of Cambridge
Cambridge CB1 8RN

**William Hamilton** W.Hamilton@exeter.ac.uk
Professor of Primary Care Diagnostics
College House, St Luke's Campus
University of Exeter
Exeter EX2 4TE

**Greg P Rubin** g.p.rubin@durham.ac.uk
Professor of General Practice and Primary Care
School of Medicine, Pharmacy & Health
Durham University
Stockton on Tees TS17 6BH

**Fiona M Walter** fmw22@medschl.cam.ac.uk
Principal Researcher in Primary Care Cancer Research
Primary Care Unit
Department of Public Health & Primary Care
University of Cambridge
Cambridge CB1 8RN

**Keywords**: pancreatic cancer, diagnosis, appraisal, help-seeking, symptoms

For peer review only

## ABSTRACT

### Objective

Pancreatic cancer has poor survival rates due to non-specific symptoms leading to later diagnosis. Understanding how patients interpret their symptoms could inform approaches to earlier diagnosis. This study sought to explore symptom appraisal and help-seeking among patients referred to secondary care for symptoms suggestive of pancreatic cancer.

### Design

Qualitative analysis of semi-structured in-depth interviews. Data were analysed iteratively and thematically, informed by the Model of Pathways to Treatment.

### Participants and setting

Pancreatic cancer occurs rarely in younger adults, therefore patients aged ≥ 40 years were recruited from nine hospitals after being referred to hospital with symptoms suggestive of pancreatic cancer; all were participants in a cohort study. Interviews were conducted soon after referral, and where possible, before diagnosis.

### Results

Twenty-six interviews were conducted (cancer n=13 (pancreas n=9, other intra-abdominal n=4), non-cancer conditions n=13; age range 48-84 years; 14 women). Time from first symptoms to first presentation to healthcare ranged from 1 to 270 days, median 21 days. We identified three main themes. Initial symptom appraisal usually began with intermittent, non-specific symptoms such as tiredness or appetite changes, attributed to diet and lifestyle, existing gastrointestinal conditions or side-effects of medication. Responses to initial symptom appraisal included changes in meal type or frequency, or self-medication. Symptom changes such as alterations in appetite and enjoyment of food or weight loss usually prompted further appraisal. Triggers to seek help included a change or worsening of symptoms, particularly pain, which was often a ‘tipping point’. Help-seeking was often encouraged by others. We found no differences in symptom appraisal and help seeking between people diagnosed with cancer and those with other conditions.

### Conclusions

Greater public and healthcare professional awareness of the combinations of subtle and intermittent symptoms, and their evolving nature, is needed to prompt timelier help-seeking and investigation among people with symptoms of pancreatic cancer.


For peer review only



For peer review only - http://bmjopen.bmj.com/site/about/guidelines.xhtml

**STRENGTHS AND LIMITATIONS OF THIS STUDY**

- We believe this is the first study to compare the symptom appraisal and help-seeking experiences of patients referred to secondary care with symptoms suggestive of pancreatic cancer between people subsequently diagnosed with pancreatic cancer and people diagnosed with other non-cancer conditions. We were unable to identify any differences.

- The data collection and analysis of this study were guided by the model of pathways to treatment as recommended by the Aarhus Statement to improve the design and reporting of studies on early cancer diagnosis.

- Risks of recall bias and post hoc rationalisation were reduced by recruiting at the time of referral to specialist care, and interviewing patients before or close to diagnosis.

- The subtlety of initial symptoms and their evolution over time is a novel finding, and provides some understanding of the complexities faced by patients when appraising their symptoms and seeking help in a timely way.

- Some of the most seriously ill patients were unable to be interviewed and their experiences may have differed from those in our sample.

For peer review only



For peer review only - http://bmjopen.bmj.com/site/about/guidelines.xhtml

## INTRODUCTION

Pancreatic cancer is the ninth most common cancer in the UK, with about 8,000 cases diagnosed every year. Only 22% men and 20% women currently survive pancreatic cancer for one year or more, and it has the poorest 5-year survival rates of all cancers at less than 4% [1]. This poor prognosis is mainly due to patients being diagnosed at a stage when curative treatment is not possible. Survival could be improved if patients could be diagnosed earlier [2,3].

The key symptoms of pancreatic cancer have primarily been described from studies of patients after a diagnosis has been made. Pre-diagnosis case-control studies from the US [4] and UK [5,6] confirm that these symptoms are also relevant for the community when making healthcare management decisions. While jaundice is the only symptom which is strongly predictive of pancreatic cancer, a number of non-specific symptoms such as nausea, vomiting, abdominal pain, back pain, constipation, diarrhoea, weight loss and malaise are also weakly associated [6]. The non-specific nature of these symptoms creates challenges for early recognition of a potentially serious condition by both patients and their GPs. In a large English database study, over 40% of patients diagnosed with pancreatic cancer had visited their GP at least three times before referral to specialist care [7].

There is limited evidence about the evolution of symptoms of pancreatic cancer over time, and how patients appraise their symptoms and decide to seek medical advice. One of the few studies to date interviewed patients or their relatives many months after diagnosis [8]; such studies may be biased by *post hoc* rationalisation and recall bias. Furthermore, many symptoms of pancreatic cancer are also symptoms of other gastrointestinal or intra-abdominal cancers such as gallbladder, colon and ovarian cancer, which may be equally difficult to diagnose early [7].

The aim of this study was therefore to use qualitative approaches to gain understanding of barriers and facilitators to symptom appraisal and help-seeking decisions among patients with symptoms suggestive of pancreatic cancer much earlier in their diagnostic pathway, to contribute to the development of interventions to promote earlier or more timely cancer diagnosis. Similar studies have been conducted for people with symptoms suggestive of lung and colorectal cancer [9-11]. We recruited patients who were newly referred to hospital, thus providing the opportunity to investigate the complexities of patients' symptom appraisal and decision making, and explore pathways from their first symptom to first presentation in primary care, emergency presentations, and referrals to specialist services.

For peer review only



For peer review only - http://bmjopen.bmj.com/site/about/guidelines.xhtml

## METHODS

### Setting

This in-depth, face-to-face interview study was nested within the SYMPTOM Pancreas Study, a prospective cohort study investigating associations between symptoms and other factors on the total diagnostic interval and stage of diagnosis among patients with symptoms suggestive of pancreatic cancer [12]. The patients were recruited from nine hospitals in two regions of England (North East and Eastern). Ethical approval for all the SYMPTOM studies was obtained from Cambridgeshire 3 Research Ethics Committee (10/H0303/50).

### Recruitment

Patients were recruited when referred to hospital via routine or urgent (two-week wait, '2WW') routes to a gastroenterology or hepato-pancreato-biliary (HPB) clinic or relevant ultrasound department, or admitted via A&E or other specialists. Pancreatic cancer occurs rarely in younger adults, therefore patients were eligible if they were aged 40 years and over, and their GPs had reported symptoms suggestive of pancreatic cancer. They were initially sent an invitation letter and SYMPTOM study questionnaire by post; people who completed the questionnaire were able to indicate whether they would be willing to take part in an interview. We purposively sampled patients (by region, age, gender, diagnosis) to obtain participants with a range of demographic and diagnosis characteristics.

### Data collection

Interviews were undertaken between March 2012 and March 2014 by KM, LB and NH, all of who have extensive qualitative research experience. They took place as soon as possible after referral to hospital.  All diagnoses were confirmed from review of secondary care medical records.

Before starting the interview, the researcher explained the research process and written consent was obtained from the participant. The interview was semi-structured, with the schedule (supplementary file 1) developed from similar interviews undertaken with people recently diagnosed with cancer [13].  By using open-ended questions we were able to explore the patient's appraisal of their symptoms and decisions to seek help.  We encouraged the interviewees to share how they made sense of their symptoms and why they chose to seek help in the context of previous experiences and existing medical conditions. To assist in the clarification of the sequences of events and key dates along the pathway to diagnosis, we used a specifically designed calendar-landmarking instrument [14].

The interviews lasted between 40-70 minutes and were conducted in participants' homes. A relative, usually a spouse, was also present at several interviews at the


For peer review only - http://bmjopen.bmj.com/site/about/guidelines.xhtml

participant's request: they sometimes assisted in recall of events and confirmed participants' accounts, but were not consented, and only the participant's words were included as data in analyses. Interviews continued until no new themes were identified in three consecutive interviews and data saturation was reached [15]. All interviews were audio-recorded, then professionally transcribed verbatim and anonymised via a confidential service.

**Analyses**

***Theoretical model***

Both data collection and analysis were underpinned by the theoretical approach of the Model of Pathways to Treatment [16,17] as recommended by the Aarhus Statement to improve the design and reporting of studies on early cancer diagnosis [18]. The Model considers the contributions of disease, patient and healthcare system factors to four intervals: Appraisal, Help-seeking, Diagnostic and Pre-Treatment. The first event is the detection of initial bodily changes, described as the "time point when a person becomes aware of body sensations or visual alterations, regardless of the meaning assigned to the change", these then develop over time to "symptoms", defined as a bodily change perceived to be abnormal. Symptom appraisal leads to perceiving a reason to seek help, and consulting with a healthcare professional. These key events represent the "time to presentation" (TTP) period. We defined TTP as the interval between the patient-reported date of first noticing a symptom and their first consultation with a healthcare professional.

***Analytic processes***

The analytic process was iterative, starting after the first few interviews. Three researchers with social sciences expertise (KM, LB, NH) worked together on an initial thematic analysis with inductive coding [19], and then adopted a deductive approach guided by the framework of the Model of Pathways to Treatment [16,17,20] to construct themes and sub-themes. These approaches were combined to ensure a rigorous and systematic progression through the five analytic steps: familiarisation with data, developing a thematic framework; indexing data to framework; mapping and questioning the data; and theoretical interpretation [21]. The Model was used at several points in the analysis process: first, to provide structure to understanding the patient pathways to diagnosis; second, to facilitate comparisons between participants diagnosed with cancer and those who presented with similar symptoms but were diagnosed with non-cancer conditions. Finally, the researchers examined patient data by characteristics, including age, gender, co-morbidity, geographical region and diagnosis, to seek patterns and non-confirming cases.

The study patient representatives (MJ, JL) have personal experiences of cancer, which was reflected in their interpretation of the data. They contributed to all stages



For peer review only - http://bmjopen.bmj.com/site/about/guidelines.xhtml

For peer review only

of analysis by reading transcripts and regular meetings with the research team, and the other authors (all with clinical or social science expertise) contributed to the interpretation of the data. Data management was assisted by NVivo version 9.

**RESULTS**

**Sample (Table 1)**

302/407 (74%) of the participants recruited into the main SYMPTOM Pancreas Study expressed an interest in an interview. Thirty-four individuals were recruited following purposive sampling, but 8 were not interviewed as they were too unwell to participate. Six were unaware of their diagnosis at interview.

Half (13/26) of the interviewees were ultimately diagnosed with cancer, 9 with pancreatic cancer (3 early stage, 3 late stage, 3 unstaged), and 4 with other cancers (duodenal, gallbladder, oesophageal, lymphoma). The 13 participants with non-cancer diagnoses included hiatus hernia, gallstones and pancreatitis. All participants were interviewed soon after their referral to specialist care (range: 1 day to 18 weeks), with a quarter (6, 23%) prior to diagnosis, half (12, 46%) within 4 weeks of diagnosis, and the remainder within 18 weeks (8, 31%).

**Duration of symptoms**

Most participants were able to recall when they first noticed their symptom/s and the date they went to their GP, though for some it was difficult to recall the exact date they decided to seek help. The TTP ranged from 1-270 days, with a median of 21 days. Three individuals were frequent attenders to their GP and so we were unable to define the precise first date of presentation. Two thirds (68%) consulted their GP within 6 weeks of their trigger symptom. While more than half (58%) consulted their GPs only once before referral to secondary care, four patients (16%) saw their GP on more than three occasions.

For peer review only



For peer review only - http://bmjopen.bmj.com/site/about/guidelines.xhtml

**Table 1: Participant characteristics**

| | Cancer diagnosis n=13 (%) | Other conditions diagnosis n= 13 (%) |
|---|---|---|
| **English region** | | |
| Eastern | 10 (77) | 9 (69) |
| North Eastern | 3 (23) | 4 (31) |
| **Gender** | | |
| Female | 6 (46) | 8 (62) |
| Male | 7(54) | 5 (38) |
| **Age** | | |
| Mean Years (range) | 67 (50-84) | 63 (49-84) |
| 40-59 | 4 (31) | 3 (23) |
| 60-69 | 4 (31) | 4 (31) |
| 70-79 | 2 (15) | 4 (31) |
| 80+ | 3(23) | 2 (15) |
| **Ethnicity** | | |
| White British | 13 (100) | 13 (100) |
| **Educational qualifications** | | |
| Higher Education | 3 (23) | 1 (8) |
| Further Education/Secondary school | 6 (46) | 8 (62) |
| None | 4 (31) | 4 (31) |
| **IMD quintiles** | | |
| Least deprived 1 | 4 (31) | 2 (15) |
| 2 | 4 (31) | 4 (31) |
| 3 | 2 (15) | 3 (23) |
| 4 | 3 (23) | 2 (15) |
| Most deprived 5 | 0 (0) | 2 (15) |
| **Diagnoses** | | |
| **Cancer** | | |
| Pancreas | 9 (69) | - |
| Duodenal | 1 (8) | - |
| Gallbladder | 1 (8) | - |
| Oesophageal | 1 (8) | - |
| Lymphoma | 1 (8) | - |
| **Other conditions** | | |
| Hiatus Hernia | - | 3 (23) |
| Gallstones | - | 2 (15) |
| Pancreatitis | - | 2 (15) |
| Others | - | 6 (46) |
| **GP appointments made** | | |
| 0* | 0 (0) | 2 (15) |
| 1 | 9 (62) | 6 (46) |
| 2 | 1 (8) | 4 (31) |
| *emergency presentation 3+ | 3 (23) | 1 (8) |



For peer review only - http://bmjopen.bmj.com/site/about/guidelines.xhtml

**Qualitative themes**

We were unable to find any clear differences regarding symptom appraisal or help-seeking between the accounts of people diagnosed with cancer and those with other conditions. We therefore report the findings together. We were also unable to find any clear differences according to whether or not participants knew their diagnosis at the time of the interview, or their characteristics including gender, age and region. Extracts from interviews illustrate the findings: each quotation is contextualised, by the patient's gender (M/F), age (40-59, 60-69, 70-79, 80+), TTP (weeks), and diagnosis (cancer, non-cancer).

<u>**Theme 1: Initial appraisal of symptoms**</u>

Two main sub-themes emerged as participants described how they first noticed and initially interpreted their symptoms, and often attributed them to a common non-serious cause or condition.

***1.1 Bodily changes***

People mainly described initial subtle or non-specific bodily changes such as feeling tired or 'different'. These changes were often intermittent and insidious in their development.

*"I've had this sort of tiredness and a little bit of discomfort on and off for a time"* (M, 70-79, 13-26 weeks, cancer)

*"I've felt a bit like as if I've got a nervous tummy, like it gurgles up…"* (F 50-59, regular attender to GP, non-cancer)

People also described subtle changes in appetite: *"Well I went off my food a bit I think, my daughter said I went off my food a bit"* (F, 80+yrs, 2-4 weeks, cancer)

***1.2 Symptom attributions (Table 2)***

Initial explanations for these bodily changes or symptoms mainly related to diet and other daily activities. Nine participants with other conditions, particularly gastro-intestinal conditions such as gastro-oesophageal reflux disease, often appraised their new symptoms against their "usual" symptoms, and whether their treatment was still effective. This group tended to downplay their symptoms. Some people also attributed these bodily changes or symptoms to other medical conditions or side-effects of their medication. Others 'normalised' their initial symptoms to being caused by aging, common conditions such as a virus or common cold, indigestion or a urine infection.

These explanations were described in the rich context of people's lives, such as being busy, caring for others, or the time of the year. When considered in these contexts, most people initially felt that their symptoms were acceptable and did not warrant seeking help:



For peer review only - http://bmjopen.bmj.com/site/about/guidelines.xhtml

*"I thought I was a bit tired-er than normal, but I mean, I was tearing around. My daughter was coming back for three months and they've got a house in the next village, I was over there doing things in the house, getting it ready."* (F, 60-69, 2-4 weeks, cancer)

**Table 2: Illustrative quotations relating to Symptom Attribution, a sub-theme of Theme 1- Initial Appraisal of Symptoms**

| | |
|---|---|
| Diet and lifestyle | *"It comes and goes, it must be something that I eat and it just doesn't agree with me"* (F, 60-69, 13-26 weeks, non-cancer)<br><br>*"I think it's the picking up, it's the bending that does it a lot with my job as well, 'cos I lift heavy (clothing) all day, and I'm putting them over people and then bending and sitting down"* (F, 40-59, regular attender to GP, cancer) |
| Existing gastrointestinal conditions | *"I've always suffered with what's it called, you know, reflux, over the years, sometimes I could take some indigestion remedy and that Gaviscon thing and it'd calm down"* (F, 40-49, <2 weeks, non-cancer) |
| Other medical conditions, side-effects of medication | *"At first I started putting it down to the diabetic pills, I thought 'Well it's probably because I've changed those', you know, and this Pioglitazone, I kept thinking 'Well it's that',* (F 60-69, 2-4 weeks, cancer)<br><br>*"I've had this sort of tiredness and a little bit of discomfort on and off for a time, I thought it was possibly diabetes…it kept on… and we thought was perhaps as a result of some of the medication I was taking for other things."* (M, 70-79, 13-26 weeks, cancer) |

<u>**Theme 2: Responses to initial symptom appraisal**</u>

Participants reported a number of responses to initial symptom appraisal.

***2.1 Lifestyle adaptations***

In the light of these attributions and explanations, some people began to make adaptations to their diet or daily living activities to alleviate their symptoms; in some cases, these changes were quite significant:

*"If I had smaller meals, but slightly more frequently, I could actually control keeping food, you know, keeping comfortable"* (M, 60-69, 5-8 weeks, cancer)

*"I think you learn to live with it and you just adapt to it and start to make the changes so it doesn't happen…. I think what had happened with me is I couldn't control it any longer, no matter if I stopped eating things, didn't eat out, it was happening anyway"* (F, 60-69, 26-52 weeks, non-cancer)

***2.2 Role of others in appraisal and adaptations***

Some people discussed their early symptoms with close relatives, particularly partners or children. This could prompt discussions around potential explanations



For peer review only - http://bmjopen.bmj.com/site/about/guidelines.xhtml

and suggestions for improving symptoms, such as changes in content or timing of meals; at times it could also lead to delays in helpseeking:
*"When I mentioned to my wife the colour of my stools, which was the first symptom, she said I wasn't getting enough carbohydrates or protein or something…something wasn't right in my diet. So I changed my diet, I was eating fruit and vegetables and things like that, a lot more, and it still stayed the same."* (M, 50-59, 13-26 weeks, cancer)

**<u>Theme 3: Further appraisal of symptoms</u>**

Changes in patterns of symptoms often led participants to further appraise their symptoms, and to question the reasons for their occurrence and their original explanations.

***3.1 Symptom progression (Table 3)***

Symptoms became of greater concern when they became more severe or frequent, or when further subtle symptoms developed. In some cases, it appeared that there was progression from subtle and intermittent symptoms of a manageable and tolerable nature, to symptoms which were more alarming such as pain. Across the whole sample, people described pain in a number of different parts of the body including their shoulder, chest, upper abdomen and back.

Another man described the chronology of his symptoms; having started with mild indigestion symptoms, additional, more alarming, symptoms developed: *"gradually… the stools over a period of time, but the urine it seemed to happen more or less in a week, from normal colour to really deep colour"* (M, 50-59, 13-26 weeks, cancer)

Many participants reported subtle yet progressive changes to their appetite and enjoyment of food. Weight loss as a result of changes in appetite was also reported by a few participants.

***3.2 Symptom monitoring and self-management***

The subtlety and slow progression of symptoms prompted some participants to monitor their symptoms, and some to consider alternative self-management strategies. Many used over-the-counter remedies to attempt to alleviate their symptoms of indigestion or abdominal pain:
*"It was only when I started feeling a bit of strangeness that I decided to try and keep a pattern of them"* (F, 60-69, 2-4 weeks, cancer)

***3.3 Impact on life***

Most people described how their symptoms gradually or rapidly impacted on their quality of life and daily routine, including their social activities and hobbies, as well as their ability to sleep. Some felt that they were less able to complete tasks at home; for others, work became more challenging:
*"I decided I'd got this burning pain and I noticed that I was beginning to get a little bit short of breath, and also I couldn't do the same things I used to, like I found digging was becoming impossible"* (M, 70-79, 13-26 weeks, non-cancer)

***3.4 Previous family and personal history of cancer***



For peer review only - http://bmjopen.bmj.com/site/about/guidelines.xhtml

A few people expressed a concern that they might develop cancer because they had lost a close relative to the disease; one had also previously suffered from cancer. These people were concerned about their personal risk of cancer and reported that they often considered whether their symptoms matched those which they expected from having cancer. Conversely one woman, subsequently diagnosed with a benign condition, had not considered cancer as a possibility because her symptoms did not match her expectations of cancer:

*"I knew I'm generally healthy, I mean I've been going swimming and to the gym…it wasn't seriously debilitating…I expect when you have cancer that you have more symptoms"* (F, 60-69, 26-52 weeks, non-cancer)

**Table 3: Illustrative quotations relating to Symptom Progression, a sub-theme of Theme 3- Further Appraisal of Symptoms**

| | |
|---|---|
| Pain | *"Well, it varied, you know, sometimes I'd have the pain all day and that's very wearing and painful…It's just under the rib cage and it's slightly to the right hand side, just slightly there, always in the same place."* (F, 70-79, <2 weeks, non-cancer)<br><br>*"…I just had a nagging, more like a dull ache, underneath my bust and I thought it was indigestion and it didn't seem to go away, it didn't make any difference whether I ate or not, it was there quite a lot of the time"* (F, 70-79, 2-4 weeks, cancer)<br><br>*"After Christmas that then became all day, every day. I was going to bed with a stomach ache and I was waking up with a stomach ache…It was there all the while and you know, it didn't make sense that I'd have a problem all the while so I thought I was imagining it"* (M, 60-69, 2-4 weeks, cancer) |
| Progressive changes in appetite and enjoyment of food | *"I was actually going off the actual taste of food, I wasn't enjoying food anymore, things that I would normally eat I wasn't enjoying"* (M, 60-69, 5-8 weeks, cancer)<br><br>*"Well it became quite traumatic at one point, when I just [did] not want an evening meal at all. I always eat a reasonable breakfast, but for the evening meal I had no appetite"* (M, 80+, <2 weeks, cancer)<br><br>*… and I was eating less and smaller meals and being really careful what I ate, and I thought "it's good for you but you shouldn't have to be that precise when you eat"* (F, 60-69, 26-52 weeks, non-cancer) |
| Weight loss | *"I was also suffering uncomfortable, you know, let's say stomach irritation, indigestion, wasn't happy eating, so I literally went along to say 'look, this isn't right, I don't mind losing weight but not like this"* (M 60-69, 5-8 weeks, cancer)<br><br>*"I'd probably lost about four pounds which was concerning me but then I thought, you've reduced the amount that you're eating…"* (F 50-59, 2-4 weeks, non-cancer) |



For peer review only - http://bmjopen.bmj.com/site/about/guidelines.xhtml

**Theme 4: Deciding to seek help (Table 4)**

The further appraisal of symptoms eventually led most participants to decide to seek help from primary care, although several had emergency visits to A&E.

***4.1 Triggers***

Triggers to seek help usually involved an increased frequency or intensity of symptoms, although sometimes it was precipitated by an alarming event, usually pain. Pain was often the "tipping point" to seek help against a background of other more subtle symptoms. For those without pain, triggers were often complex, and could involve interpreting new or changing symptoms alongside symptoms from other existing conditions and, in some instances, symptoms from other acute illnesses. Healthcare factors, such as availability of a GP appointment, were seldom mentioned.

***4.2 Legitimising help-seeking***

People were often encouraged to seek help by their spouse, family members and friends; they were particularly influenced by comments about changes in their general wellbeing, or recall of specific symptom episodes. A few participants reported considering whether their symptoms had become a 'legitimate' reason to seek healthcare when self-management strategies became ineffective; they tended to refer to the length of time symptoms had been experienced, and their reduced ability to cope.

**Table 4: Illustrative quotations relating to Theme 4: Deciding to Seek Help**

| | Illustrative quotations |
|---|---|
| Pain on a background of more subtle symptoms | *"Sometimes I could take some indigestion remedy and.. it'd calm down, but this were a different kind of pain, this were, oh it's excruciating pain…but that were the first time that… I thought there's something not right here and I need to get some help."* (F, 40-49, <2 weeks, non-cancer) |
| Help-seeking encouraged by others | *"The kids had been saying "mum you're always ill" and I thought "oh yeah, I am". Because you, I think you sort of drift, you get used to things and you don't see any problem with it, and when other people start to say, you know "hang on" and then I thought "oh yeah"*(F, 60-69, 26-52 weeks, non-cancer)<br><br>*"I'd fall asleep on my desk, you know, this isn't me, and other people said "oh you're losing weight"… people actually were saying "you don't look well". It's another lever to go and see, to get it sorted"* (M, 60-69, 5-8 weeks, cancer) |
| Legitimate reason | *"I think my basic problem was pain… from arthritis that I'd had for some time, but it was proving impossible to control it by the methods I was using"* (M 80-89, 2-4 weeks, cancer) |

**DISCUSSION**



For peer review only - http://bmjopen.bmj.com/site/about/guidelines.xhtml

This is the first study to investigate symptom appraisal and help-seeking in patients newly referred to hospital with symptoms suggestive of pancreatic cancer. Due to their subtle and intermittent pattern, many people initially just monitored their symptoms. Symptoms were interpreted based on previous experiences with common conditions such as gastroenteritis, or impact of dietary change or existing gastro-intestinal conditions. Many made changes to their eating patterns including type of food, and frequency and quantity of meals, or managed their symptoms with over-the-counter medication. Consequently, they tended not to go to their GP at that point. Changes to symptom frequency, duration or severity, their impact on daily life, or the appearance of additional symptoms, particularly pain, led many people to further appraise their symptoms and visit their GP. Family and friends often made important contributions to encouraging help-seeking by reflecting on changes in symptoms, although at times social contacts could re-inforce self-management strategies. These patterns of symptom appraisal and help-seeking were seen regardless of final diagnosis.

**Strengths and limitations**

The major strength of this study is that we prospectively recruited patients with symptoms suggestive of pancreatic cancer, and interviewed them during their pathway to diagnosis and treatment. This allowed detailed discussions about factors affecting symptom appraisal and help-seeking while they were still fresh events, and greatly reduced the possibility of *post-hoc* rationalisation and recall bias. We have used similar approaches in interview studies with people recently diagnosed with melanoma [22], and people with symptoms suggestive of lung [10] and colorectal [12] cancers. Recruiting patients during their diagnostic pathway enabled comparisons about symptom appraisal and help-seeking between people subsequently diagnosed with cancer and people with non-cancer conditions; importantly, we were not able to find differences between them. Our recruitment process meant that some patients were subsequently diagnosed with a cancer other than pancreatic cancer. We do not consider this a weakness of the study; rather, it serves to highlight the complexity and challenges of diagnosing pancreatic cancer, and how similarly a range of upper gastro-intestinal cancers can present. While we aimed to recruit equal numbers of patients with pancreatic cancer and benign conditions, several patients with cancer withdrew before interview as they were too unwell.

Our approach to data collection and analysis was strengthened by the use of a strong theoretical framework: underpinned by recommendations from the Aarhus statement on improving design and reporting studies on early cancer diagnosis [18], we applied the framework of the widely used Model of Pathways to Treatment [16,17], and a specifically-developed calendar-landmarking instrument which aided



For peer review only - http://bmjopen.bmj.com/site/about/guidelines.xhtml

some participants' recall of dates of their symptoms and help-seeking [14]. Other strengths include recruitment from two English regions and a number of hospitals to ensure patients from a wide range of socio-economic backgrounds, and who had entered hospital care via a variety of routes, enabling us to gather a broad range of experiences. Finally, the research team had a broad range of scientific and clinical expertise, and we sought input from our lay members at all stages of the research process, including analysis and interpretation of the data.

While we acknowledge that these experiences may not be representative of all people with symptoms suggestive of pancreatic cancer, the demographics of this cohort are similar to that of the main SYMPTOM-Pancreas study [12]. Although a major finding was that there were similar patterns in symptom appraisal and help-seeking between those diagnosed with cancer and those with other diagnoses, this may have been partly due to recruiting patients relatively late in their diagnostic pathway, when GPs had referred with a suspicion of pancreatic cancer. Ideally we would have recruited people with similar symptoms from earlier in their diagnostic pathway and from primary care, but there are significant challenges with the feasibility and costs of recruiting such a cohort containing sufficient cases of pancreatic cancer. The presence of relatives in some interviews may have led to potential bias in the participants' accounts and recollections, but this was minimised by only including participant's words in the analysis. Finally, we can only report the experiences shared with the researchers at the time of the interview: there may have been events which were not discussed if they were felt to be too emotional or private.

**Comparison with existing literature**

Although these findings are in line with the broader literature investigating help-seeking for cancer symptoms [10,12, 22], very little has been published on symptom appraisal and help-seeking among patients diagnosed with pancreatic cancer. Evans *et al.* recently reported the only other similar study, also set in England, and highlighted the intermittent and subtle nature of symptoms with their title: '"It can't be very important because it comes and goes"- patients' accounts of intermittent symptoms preceding a pancreatic cancer diagnosis' [8]. However, their interviews were conducted with people up to several years after a diagnosis of pancreatic cancer, and included data from interviews with relatives of people who had died. Our findings are strengthened by interviewing patients earlier in their illness pathway, thereby capturing subtle assessments of initial bodily changes and explanations, and minimising post-hoc rationalisation and recall bias. We could not identify any subtle differences in the nature of symptoms or help-seeking between those who eventually were found to have pancreatic cancer, and those who were not. This is consistent with the quantitative findings of the SYMPTOM-pancreas study



For peer review only - http://bmjopen.bmj.com/site/about/guidelines.xhtml

[9], where the evolution of additional symptoms such as fatigue, change in bowel habit, weight loss, decreased appetite and jaundice were associated with pancreatic cancer.

Other studies of pancreatic cancer symptoms have mainly used quantitative retrospective designs. For example, Stapley *et al*. undertook a case-control study using routinely recorded UK primary care data which demonstrated how poorly predictive most symptoms are of pancreatic cancer in primary care populations. While the positive predictive value of jaundice was relatively high (21%), it was much lower for other symptoms including abdominal and back pain, nausea, vomiting, change in bowel habit, weight loss and malaise (<1%) [6]. Keane *et al*. more recently used another large UK primary care database to investigate the early symptom profiles of patients diagnosed with pancreatic ductal or biliary adenocarcinoma and found a similar wide range of early symptoms [23]. They also found that, in the year prior to diagnosis, these patients visited their GP on a median of 18 (IQR 11–27) occasions, which resonated with findings from another large English database study showing that 41% of patients diagnosed with pancreatic cancer had visited their GP at least three times before referral to specialist care [7]. In contrast, more than half our sample reported only visiting their GP once before referral, suggesting either that our sample were recruited early in their symptom pathway, or perhaps that they had symptoms such as jaundice which are more likely to prompt early referral. Other quantitative studies set among patients newly diagnosed with pancreatic cancer patients have reported fatigue, dyspepsia and subtle changes in appetite such as early satiety in the preceding six months, confirming our finding that there may be a window of opportunity for timely investigations when patients report combinations of non-specific or subtle symptoms [4,24,25].

**Implications for research and practice**

Our findings add to current knowledge about the subtle and non-specific nature of early symptoms of pancreatic cancer, and enhance our understanding of their often intermittent and insidious nature. They highlight the importance of on-going, and often iterative, appraisal of symptoms as described in the Model of Pathways to Treatment [16,17]. The findings have characterised pain as a 'tipping point' for presentation to healthcare, and underpinned the importance of family and friends in symptom monitoring, recognising evolving and new symptoms and endorsement of help-seeking [26]. The challenge for symptom awareness campaigns is to promote earlier presentation with these more subtle and intermittent symptoms which lead to changes in eating patterns including type of food, and frequency and quantity of meals, and weight loss. The 'Know 4 Sure' campaign was one of the first to raise awareness of non-site-specific symptoms including weight loss, pain, lump and unexplained bleeding. It was associated with increase in referrals for a range of



For peer review only - http://bmjopen.bmj.com/site/about/guidelines.xhtml

cancers including upper GI cancer [27]. Whether public health campaigns can effectively promote messages about combinations of non-specific symptoms is unknown, but may be important in pancreatic cancer.

The newly revised NICE guidance on Suspected Cancer: Recognition and Referral [28] supports a lower threshold for investigating and referring people with single and multiple symptoms suggestive of pancreatic cancer, particularly those in higher risk groups such as newly diagnosed diabetics. The findings from this study could inform GP education approaches to promote timely investigation and referral for people with these non-specific and intermittent symptoms. These numerous subtle and non-specific symptoms, when combined with abnormal blood tests and the use of clinical decision support tools, might alert GPs to a possible pancreatic cancer diagnosis earlier. Safety-netting, defined as is a diagnostic strategy or consultation technique to help manage diagnostic uncertainty and ensure that patients are followed up in a timely and appropriate manner, is also important due to the evolving nature of symptoms; appropriate strategies would include symptom monitoring and early review.

In conclusion, this study has described and contextualised the appraisal and help-seeking behaviours of patients with symptoms that could represent pancreatic cancer. Greater awareness of combinations of the subtle and intermittent symptoms, and their evolving nature, is needed to prompt timelier help-seeking and investigation among people with symptoms of pancreatic cancer.

For peer review only



For peer review only - http://bmjopen.bmj.com/site/about/guidelines.xhtml

**Acknowledgments**

We thank the patients who so kindly and freely gave their time and experiences to this study. We thank Helen Morris the SYMPTOM study manager, our other study patient and public representatives Sue Ballard and Victor Boulter, and the staff at the participating hospitals (Bedford Hospital NHS Trust, Cambridge University Hospitals NHS Foundation Trust; Hinchingbooke Healthcare NHS Trust, The Queen Elizabeth Hospital Kings Lynn NHS Trust, Peterborough and Stamford NHS Foundation Trust, University Hospital of North Durham; Darlington Memorial Hospital: University Hospital of North Tees, West Suffolk NHS Foundation Trust) who facilitated participant recruitment and supported the study. The authors would also like to acknowledge the contribution made by the NIHR funded Discovery Programme Steering Committee comprising: Roger Jones (chair); Alison Clutterbuck; Ardiana Gjini; Joanne Hartland; Maire Justice; Jenny Knowles; Richard Neal; Peter Rose.

**Contributors**

FMW, JE, GR and WH were responsible for study design, study conduct and management. KM, LB and NH collected the data, and with the support of JB, JE, JL, MJ and FMW, KM analysed and interpreted the data. KM led on the manuscript preparation. All authors contributed to the writing and completion of the paper and have approved the final the manuscript.

**Funding**

This paper presents independent research funded by the National Institute for Health Research Programme Grants for Applied Research programme (RP-PG-0608-10045) and Pancreatic Cancer Action (registered charity 1137689). The views expressed are those of the authors and not necessarily those of the NHS, the NIHR or the Department of Health. The funders of this study had no role in study design, data collection, analysis, and interpretation, or the writing of this research paper. FMW is supported by an NIHR Clinician Scientist award.

**Competing interests**

The authors have no conflicts of interest to declare.

**Data sharing statement**

No additional data are available.

**Ethics approval**

Ethical approval was granted by the Cambridgeshire 3 Research Ethics Committee (10/H0306/50).

For peer review only

**REFERENCES**

1. Cancer Research UK, *Incidence Statistics* 2011. Retrieved from http://www.cancerresearchuk.org/health-professional/cancer-statistics/incidence/common-cancers-compared#heading-Zero (Last accessed 31.05.16).

2. Hiom S. Diagnosing cancer earlier: reviewing the evidence for improving cancer survival. *Br J Cancer* 2015; 112 (supp1): S1-S5.

3. Neal RD, Tharmanathan P, France B et al. Is increased time to diagnosis and treatment in symptomatic cancer associated with poorer outcomes? Systematic review. Br J Cancer 2015; 112:S92-S107.

4. Holly EA, Chaliha I, Bracci PM, et al. Signs and symptoms of pancreatic cancer: a population-based case-control study in the San Francisco Bay area. *Clin Gastroenterol Hepatol* 2004; 2:510-7.

5. Hippisley-Cox J, Coupland C. Identifying patients with suspected pancreatic cancer in primary care: derivation and validation of an algorithm. *Br J Gen Pract* 2012; 62: e38-45.

6. Stapley S, Peters TJ, Neal RD, et al. The risk of pancreatic cancer in symptomatic patients in primary care: a large case-control study using electronic records. *Br J Cancer* 2012; 106: 1940-4.

7. Lyratzopoulos G, Neal RD, Barbiere JM, et al. Variation in number of general practitioner consultations before hospital referral for cancer: findings from the 2010 National Cancer Patient Experience Survey in England, *The Lancet Oncology* 2012; 13: 353–365.

8. Evans J, Chapple A, Salisbury H, et al. "It can't be very important because it comes and goes"--patients' accounts of intermittent symptoms preceding a pancreatic cancer diagnosis: a qualitative study. *BMJ Open* 2014; 4: e004215.

9. Birt L, Hall N, Emery J, et al. Responding to symptoms suggestive of lung cancer: a qualitative interview study. *BMJ Open Respir Res* 2014; 1(1): e000067.

10. Walter FM, Rubin G, Bankhead C, et al. Symptoms and other factors associated with time to diagnosis and stage of lung cancer: a prospective cohort study. *Br J Cancer* 2015; 112 (supp1): S6-S13.

11. Hall N, Birt L, Banks J et al. Symptom appraisal and healthcare-seeking for symptoms suggestive of colorectal cancer: a qualitative study. *BMJ Open* 2015;5(10):e008448.

12. Walter FM, Mills K, Mendonca SC, et al. Symptoms and patient factors associated with diagnostic intervals for pancreatic cancer (SYMPTOM pancreatic study): a prospective cohort study. *Lancet Gastroenterol Hepatol* 2016; 1:298-306.



For peer review only - http://bmjopen.bmj.com/site/about/guidelines.xhtml

13. Emery JD, Walter FM, Gray V, et al. Diagnosing cancer in the bush: a mixed methods study of GP and specialist diagnostic intervals in rural Western Australia. *Fam Pract* 2013; 30(5):541-50.

14. Mills K, Emery J, Cheung C, et al. A qualitative exploration of the use of calendar landmarking instruments in cancer symptom research. BMC *Fam Pract* 2014; 15:167.

15. Guest G, Bunce A, Johnson L. How many interviews are enough? An experiment in data saturation and variability. *Field Methods* 2006; 18:59-82.

16. Walter FM, Scott SE, Webster A, et al. The Andersen Model of Total Patient Delay: a systematic review of its application in cancer diagnosis. *J Health Services Research & Policy* 2012; 17(2):110-8.

17. Scott SE, Walter FM, Webster A et al. The model of pathways to treatment: conceptualization and integration with existing theory. *Br J Health Psychol* 2013; 18(1): 45-65.

18. Weller D, Vedsted P, Rubin G, et al. The Aarhus statement: improving design and reporting of studies on early cancer diagnosis. *Br J Cancer* 2012; 106(7):1262-1267.

19. Braun, V. and Clarke, V. Using thematic analysis in psychology. Qualitative Research in Psychology, 2006, 3 (2). pp. 77-101.

20. Gale NK, Heath G, Cameron E, Rashid S, Redwood S. Using the framework method for the analysis of qualitative data in multi-disciplinary health research, BMC Med Res Methodol. 2013 Sep 18;13:117.

21. Ritchie J, Spencer L. Qualitative data analysis for applied policy research. In: Bryman A, Burgess R, eds. *Analysing qualitative data*. London: Routledge, 1993:173-94.

22. Walter FM, Birt L, Cavers D, et al. This isn't what mine looked like: a qualitative study of symptom appraisal and help-seeking in people recently diagnosed with melanoma. *BMJ Open* 2014; 21: 4(7) e005566.

23. Keane MG, Horsfall L, Rait G, Pereira SP. A case-control study comparing the incidence of early symptoms in pancreatic and biliary tract cancer. *BMJ Open* 4:e005720.

24. Gullo L, Tomassetti P, Migliori M, et al. Do early symptoms of pancreatic cancer exist that can allow an earlier diagnosis? *Pancreas* 2001; 22:210-3.

25. Ingeman ML, Christensen MB, Bro F. The Danish cancer pathway for patients with serious non-specific symptoms and signs of cancer–a cross-sectional study of patient characteristics and cancer probability, *BMC Cancer* 2015; 15:421.

For peer review only



For peer review only - http://bmjopen.bmj.com/site/about/guidelines.xhtml

26. Leydon GM, Bynoe-Sutherland J, Coleman MP. The journey towards a cancer diagnosis: the experiences of people with cancer, their family and carers. *Eur J Cancer Care* 2003; 12:317-326.

27. Cancer Research UK, "Know 4 sure" campaign 2014. Retrieved from http://www.cancerresearchuk.org/health-professional/early-diagnosis-activities/be-clear-on-cancer/know-4-sure-campaign (Last accessed 31.05.2016).

28. National Institute for Health and Care Excellence, Suspected Cancer: Recognition and Referral 2015. Retrieved from http://www.nice.org.uk/guidance/ng12 (Last accessed 31.05.16).

For peer review only



For peer review only - http://bmjopen.bmj.com/site/about/guidelines.xhtml

# The SYMPTOM Study

## Interview Topic Guide

1. Welcome and introduction

2. Confirm understanding of study, participation & recording, confidentiality issues & consent

3. Can you tell me about what first prompted you to go to your doctor?

4. Can you describe the symptom(s) a bit more?
   *[Prompts: location, duration, severity, ever had this before?]*

5. You mentioned (symptom(s)), were there any more symptoms that you noticed about this time?
   *[Prompt: try to identify all participant-reported symptoms first; the checklist comes later]*

6. What did you do to cope with/help these symptoms?
   *[Prompts: How long did it take before you decided to use any treatment? How long did you try it for? Did it help at all?]*

7. So, please could you help me try and record the number and chronology/timing of each symptom?
   a. *Using the Calendar landmarking tool (see below)*
   b. *By drawing a timeline of each symptom with the participant*
   c. *By using the model (attached) to identify appraisal, help-seeking, and diagnostic intervals.*

7. Now, we've talked about the symptoms that you have described: I'd also like to check whether you have had any of the following symptoms?
   *[Prompt for checklist, and try to draw the timeline for each symptom: Lung symptoms: e.g. coughing up blood, dry/productive cough, chest tightness/pain, hoarse voice etc.; Colorectal & pancreas symptoms: e.g. abdominal pain, change of bowel habit, rectal bleeding, yellow jaundice etc.; General symptoms: e.g. increased fatigue, loss of appetite, weight loss etc.]*

9. You went to see your doctor about your symptoms: did you have any thoughts about what might have been wrong?

10. Did you discuss your symptoms with anyone other than your Doctor?
    *[Prompts: Who were they? Did they encourage/discourage you to seek medical advice?]*

11. Do you know of anyone who has also had any similar symptoms or investigations to you?
    *[Prompts: relationship, symptoms they had, care they received, outcome etc]*

12. Can you tell me about the first appointment with your doctor for these symptoms we've been talking about?
    *[Prompts: Was there a specific event that made you make the appointment? What was the trigger? Had you waited for any reason?]*

13. What was discussed at that first appointment?
    *[Prompts: Did you have any thoughts about what would happen when you first went to see your GP? What happened– were you examined? Did the GP arrange any tests or referrals?]*

14. How did you feel about the outcome of the consultation?
    *[Prompt: did you feel you were taken seriously by the GP?]*

15. Did you see the GP for this symptom more than once before you were referred to the

For peer review only


For peer review only - http://bmjopen.bmj.com/site/about/guidelines.xhtml

hospital?
*[Prompt: each visit may need unpacking for symptoms discussed, feelings about the consultation outcomes etc.]*

*16.* In your opinion, do you feel there was anything that prolonged you finding out about what is causing your symptoms [*Prompts: information about the symptoms associated with serious conditions, difficulty in getting an appointment, time stresses from work/family etc., medical staff attitudes etc.]*

17. Have you experienced any symptoms or feelings or sensations that you *didn't* talk to the GP about, even if you just felt "a bit off".
*[Prompt: Was there a reason why you did not mention this to your doctor?]*

*18. Now we've discussed your symptoms, I'd like to ask you a few questions about being referred and the test[s] you've had. Thinking back to your first consultation with your doctor, can you tell me what tests or investigations were mentioned (CXR, colonoscopy etc). When you were referred for the test can you tell me-?*

A. What information the GP provided about this test
  - *[Prompts: info provided – face to face, info leaflet, website]*

B. Did you understand why you were being referred/tested and what it involved?
  - *[Prompts: knowledge of physical aspects of test, possible side effects, meaning of results - diagnosis, necessity of further tests]*

C. Do you feel that you made a conscious decision to have this test or do you feel that the decision was made for you by the GP
  - *[Prompts: feel involved in decision to be tested, conscious decision – why?]*
  - *[Prompts: accepted referral – why?; Conscious decision – why?]*
  - *[Prompts: consider not having test?]*
  - *[Prompts: discuss test with family/friends – did this influence?]*

D. How did you feel about being referred for this test
  - *[Prompts: advantages - improved prognosis, wider treatment options, reassurance; disadvantages - physical & emotional challenges, practicalities, any other aspects]*

E. Can you tell us what happened during this test
  - *[Prompts: Did you take a family member/ friend with you? what happened after, how information was communicated, how test differed from expectations]*

F. Can you tell us about the practicalities of taking this test?
  - *[Prompts: time off work – how long, manage family commitments & childcare]*

G. Do you think that all patients with symptoms like yours should have these tests?

19. We've been through a lot today but is there anything else you'd like to discuss?

20. Thanks, confirmation of consent and confidentiality.


For peer review only - http://bmjopen.bmj.com/site/about/guidelines.xhtml

# Consolidated criteria for reporting qualitative studies (COREQ): 32-item checklist

Developed from:
Tong A, Sainsbury P, Craig J. Consolidated criteria for reporting qualitative research (COREQ): a 32-item checklist for interviews and focus groups. *International Journal for Quality in Health Care*. 2007. Volume 19, Number 6: pp. 349 – 357

**YOU MUST PROVIDE A RESPONSE FOR ALL ITEMS. ENTER N/A IF NOT APPLICABLE**

| No. Item | Guide questions/description | Reported on Page # |
|---|---|---|
| **Domain 1: Research team and reflexivity** | | |
| *Personal Characteristics* | | |
| 1. Interviewer/facilitator | Which author/s conducted the interview or focus group? | Undertaken by K Mills, N Hall and L Birt –data collection section reported p6. |
| 2. Credentials | What were the researcher's credentials? E.g. PhD, MD | All have PhD and extensive qualitative research experience, see p6. |
| 3. Occupation | What was their occupation at the time of the study? | All were Research Associates, see p1-2. |
| 4. Gender | Was the researcher male or female? | All female. |
| 5. Experience and training | What experience or training did the researcher have? | See p6. |
| *Relationship with participants* | | |
| 6. Relationship established | Was a relationship established prior to study commencement? | No. |
| 7. Participant knowledge of the interviewer | What did the participants know about the researcher? e.g. personal goals, reasons for doing the research | Participants had received the Patient Information Sheet, see p6. |
| 8. Interviewer characteristics | What characteristics were reported about the interviewer/facilitator? e.g. Bias, assumptions, reasons and interests in the research topic | Reported under Methods, analytic processes, see p7. |
| **Domain 2: study design** | | |
| *Theoretical framework* | | |
| 9. Methodological orientation and Theory | What methodological orientation was stated to underpin the study? e.g. grounded theory, discourse analysis, | We used thematic analysis, see p7. |

For peer review only

| | | |
|---|---|---|
| | ethnography, phenomenology, content analysis | |
| *Participant selection* | | |
| 10. Sampling | How were participants selected? e.g. purposive, convenience, consecutive, snowball | Using purposive sampling, see p8. |
| 11. Method of approach | How were participants approached? e.g. face-to-face, telephone, mail, email | Recent referral to hospital, reported under recruitment section, see p6. |
| 12. Sample size | How many participants were in the study? | 26 interviews, reported on p8. |
| 13. Non-participation | How many people refused to participate or dropped out? Reasons? | 34 individuals were purposefully sampled and invited to participate. 8 were not interviewed as they were not well enough to complete the interview, described on p8. |
| *Setting* | | |
| 14. Setting of data collection | Where was the data collected? e.g. home, clinic, workplace | Home, reported on p6. |
| 15. Presence of non-participants | Was anyone else present besides the participants and researchers? | Relatives were present in a few of the interviews, reported on p6-7. |
| 16. Description of sample | What are the important characteristics of the sample? e.g. demographic data, date | See Table 1. |
| *Data collection* | | |
| 17. Interview guide | Were questions, prompts, guides provided by the authors? Was it pilot tested? | Topic guide used, similar to that used for the other SYMPTOM studies with participants with symptoms suggestive of lung and colorectal cancer (appendix) |
| 18. Repeat interviews | Were repeat inter views carried out? If yes, how many? | No |
| 19. Audio/visual recording | Did the research use audio or visual recording to collect the data? | Yes, audio-recording, see p7. |
| 20. Field notes | Were field notes made during and/or after the inter view or focus group? | Personal notes were kept, but |

For peer review only

| | | |
|---|---|---|
| | | they were not included in the analysis. |
| 21. Duration | What was the duration of the inter views or focus group? | 40-70 minutes, see p6. |
| 22. Data saturation | Was data saturation discussed? | Yes, see p7. |
| 23. Transcripts returned | Were transcripts returned to participants for comment and/or correction? | No, the authors did not consider respondent validation to be an appropriate approach in this patient group. |
| **Domain 3: analysis and findings** | | |
| *Data analysis* | | |
| 24. Number of data coders | How many data coders coded the data? | Three- KM, NH & LM coded the data, see p6. |
| 25. Description of the coding tree | Did authors provide a description of the coding tree? | No |

| | | |
|---|---|---|
| 26. Derivation of themes | Were themes identified in advance or derived from the data? | Initially inductively using data-driven coding, then deductively, using the framework of the Model of Pathways to Treatment, see p7. |
| 27. Software | What software, if applicable, was used to manage the data? | NVivo- reported on p8. |
| 28. Participant checking | Did participants provide feedback on the findings? | No, although some validation was provided by Steering group including 2 lay members who have been included as authors, reported on p7. |
| *Reporting* | | |
| 29. Quotations presented | Were participant quotations presented to illustrate the themes/findings? Was each quotation identified? e.g. participant number | Yes throughout results in the text and in Tables 2,3, and 4, reported pp 10-14. |

For peer review only

| | | |
|---|---|---|
| 30. Data and findings consistent | Was there consistency between the data presented and the findings? | Yes. |
| 31. Clarity of major themes | Were major themes clearly presented in the findings? | Yes, see pp10-14. |
| 32. Clarity of minor themes | Is there a description of diverse cases or discussion of minor themes? | Yes, see pp 10-14. |

**Once you have completed this checklist, please save a copy and upload it as part of your submission. When requested to do so as part of the upload process, please select the file type: *Checklist*. You will NOT be able to proceed with submission unless the checklist has been uploaded. Please DO NOT include this checklist as part of the main manuscript document. It must be uploaded as a separate file.**

For peer review only
